# Systematic pan-cancer analysis of somatic allele frequency

**DOI:** 10.1038/s41598-018-25462-0

**Published:** 2018-05-16

**Authors:** Liam Spurr, Muzi Li, Nawaf Alomran, Qianqian Zhang, Paula Restrepo, Mercedeh Movassagh, Chris Trenkov, Nerissa Tunnessen, Tatiyana Apanasovich, Keith A. Crandall, Nathan Edwards, Anelia Horvath

**Affiliations:** 1 Department of Pharmacology and Physiology, School of Medicine and Health Sciences, The George Washington University, Washington, DC 20037 USA; 2 McCormick Genomics and Proteomics Center, School of Medicine and Health Sciences, The George Washington University, Washington, DC 20037 USA; 3 Department of Biochemistry and Molecular and Cellular Biology, Georgetown University, School of Medicine, Washington, DC 20057 USA; 4 Department of Biochemistry and Molecular Medicine, School of Medicine and Health Sciences, The George Washington University, Washington, DC 20037 USA; 5 University of Massachusetts Medical School, Program in Bioinformatics and Integrative Biology, Worcester, MA 01605 USA; 6 Department of Statistics, The George Washington University, Washington, DC 20037 USA; 7 Computational Biology Institute, Milken Institute School of Public Health, The George Washington University, Washington, DC 20052 USA

## Abstract

Imbalanced expression of somatic alleles in cancer can suggest functional and selective features, and can therefore indicate possible driving potential of the underlying genetic variants. To explore the correlation between allele frequency of somatic variants and total gene expression of their harboring gene, we used the unique data set of matched tumor and normal RNA and DNA sequencing data of 5523 distinct single nucleotide variants in 381 individuals across 10 cancer types obtained from The Cancer Genome Atlas (TCGA). We analyzed the allele frequency in the context of the variant and gene functional features and linked it with changes in the total gene expression. We documented higher allele frequency of somatic variants in cancer-implicated genes (Cancer Gene Census, CGC). Furthermore, somatic alleles bearing premature terminating variants (PTVs), when positioned in CGC genes, appeared to be less frequently degraded via nonsense-mediated mRNA decay, indicating possible favoring of truncated proteins by the tumor transcriptome. Among the genes with multiple PTVs with high allele frequency, *ARID1*, *TP53* and *NSD1* were known key cancer genes. All together, our analyses suggest that high allele frequency of tumor somatic variants can indicate driving functionality and can serve to identify potential cancer-implicated genes.

## Introduction

Somatic genetic variants can express their effects both directly, by affecting the dynamics and efficiency of the transcript generation and degradation processes, and indirectly, through introduction of functional features that are subject to positive or negative selection. Both types of effects are likely to impact the abundance of the variant-bearing allele in the tumor transcriptome. As such, the tumor transcriptome is quickly emerging as an informative source for exploring the functionality of the somatic variants^1–3^.

Single nucleotide variations (SNVs) comprise a major fraction of the somatic alterations in the tumor genome and are a significant contributor to the tumor cell phenotype and functionality. The functional consequences of the SNVs are inferred from their predicted effect on the protein, such as amino acid change, splicing alteration, or premature truncation of the protein. The later represents a special case expected to affect the allele prevalence through depletion of the variant-bearing transcripts via nonsense-mediated mRNA decay (NMD)^4^. In addition, SNVs can express their functionality through altering sequence motifs recognizable by other molecules, such as transcription or splicing factors, or stabilizing and supporting complexes^5,6^. These effects commonly manifest in a cis fashion and directly impact, positively or negatively, the relative abundance of the variant bearing allele^5–7^.

Imbalanced somatic allele prevalence can both cause and result from altered cellular functioning and can thereby play a substantial role in cancer initiation and progression. Indeed, asymmetrically expressed alleles are reported to play a role in variety of cancer types, including blood, breast, ovarian, and lung cancer^8–12^. Importantly, several recent studies have suggested distinct patterns of allele expression for genes implicated in cancer^1–3^. This, in turn, suggests that asymmetric alleles can be used to indicate potential cancer-implicated functionality.

Several factors are critical for the assessment of somatic allele abundance and its downstream effects on cellular function. Among them, of ultimate importance is the corresponding DNA’s allele content, which reflects both copy number alterations (CNAs) and admixture with non-tumor genomes. The latter is commonly referred as genome (or sample) “purity”, and is acknowledged to impact the outcome of cancer genomic studies, with consequences on downstream analyses and results’ interpretation^13^. Second, variant-independent imprinting effects need to be distinguished in order to identify variant-specific allele preferences^14,15^. Finally, the effects of the asymmetrically expressed alleles on total gene expression are essential for assessing the downstream consequences of somatic variants. To account for all three factors in our analyses, we used the unique data set that consisted of matched tumor and normal RNA and DNA sequencing data from the same patient. By using this approach, we aimed to: (1) account for the contribution of DNA in the quantitation of the allele abundance, and (2) link allele frequency of somatic variants to total gene expression, as measured by direct assessment of the fold-change of the expression levels as compared to the matched normal tissue.

Herein, we report the results from the analysis on the allele-specific and total gene expression of somatic variants across 10 cancer types obtained through The Cancer Genome Atlas (TCGA)^16^: Urothelial Bladder Carcinoma (BLCA), Breast Invasive Carcinoma (BRCA), Head and Neck Squamous Cell Carcinoma (HNSC), Kidney Renal Clear Cell Carcinoma (KIRC), Liver Hepatocellular Carcinoma (LIHC), Lung Adenocarcinoma (LUAD), Lung Squamous Cell Carcinoma (LUSC), Prostate Adenocarcinoma (PRAD), Thyroid Carcinoma (THCA), and Uterine Corpus Endometrial Carcinoma (UCEC). We estimate allele prevalence and test for correlation with functional features, including predicted effects on the protein, location in motifs involved in interaction with other molecules, and conservation of the genome position. Next, we link somatic allele frequency to gene expression levels, as compared to the corresponding normal tissue sample. We then compare the observations between genes known to be implicated in cancer (Cancer Gene Census, CGC)^17^ and the rest of the genome, and document several patterns that are frequently confined to the CGC genes. In summary, we present an expanded set of somatic allele features, whose integrated analysis suggests novel links between transcriptome allele preference and cancer-implicated functionality.

## Results

### Analytical pipeline and overall dataset characteristics

**Figure 1:**
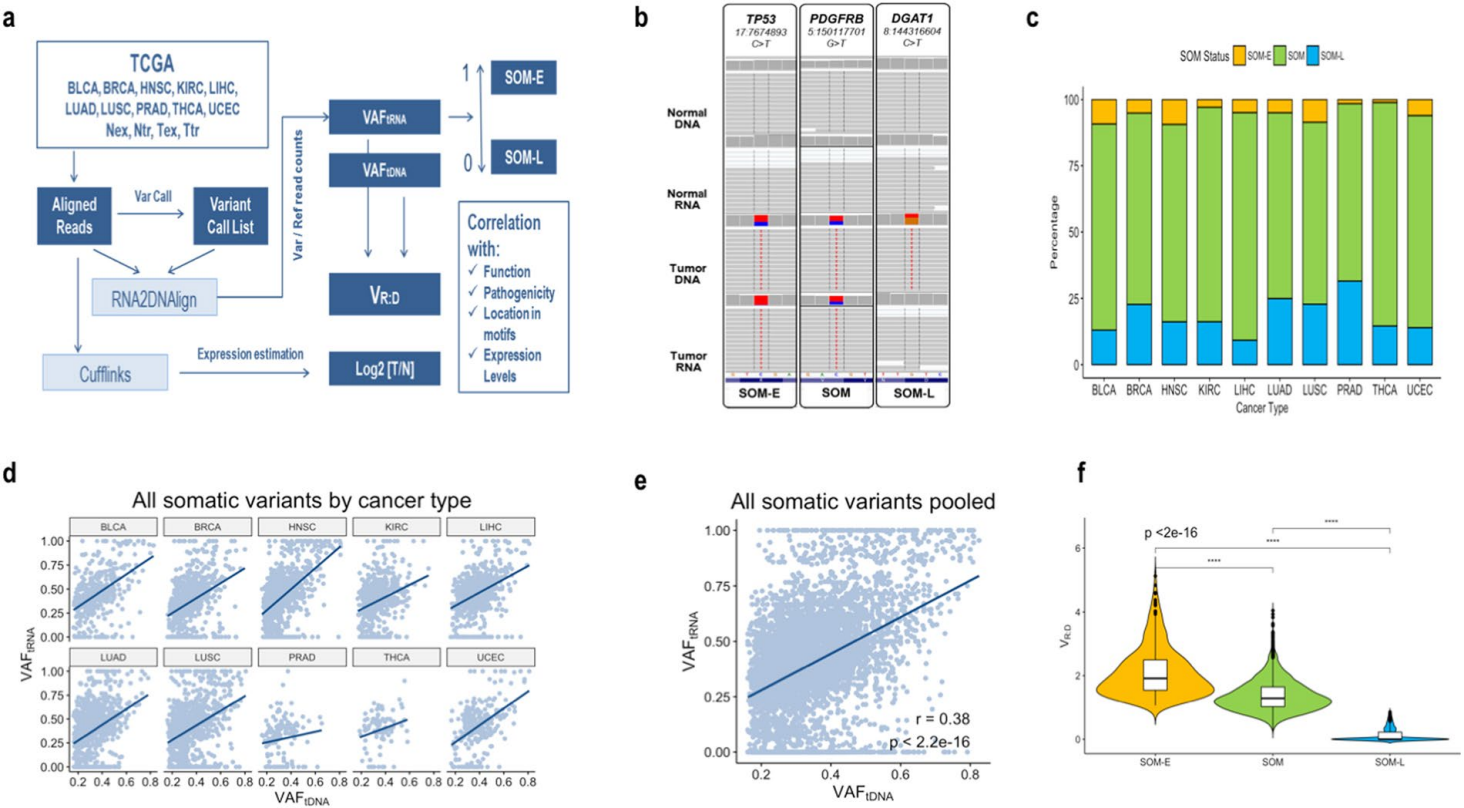
(**a)** Major steps of the analysis of allele distribution for somatic variants in the ten studied cancer types. Variant allele frequency (V_R:D_) was analyzed for correlation with different functional features and expression levels in cancer implicated genes (CGC) and the rest of the genome. SOM-E and SOM-L represent the extremes of the VAF_tRNA_. (**b**) Visualization of alignments (Integrative Genome Viewer, IGV) of examples of (from left to right) SOM-E, SOM and SOM-L variants. The bi-allelic position is reflected through color-coding of the summary flag on the top of each panel. The gray lines represent sequencing reads, and the colored letters show differences from the reference. (**c**) Proportion of SOM, SOM-E and SOM-L across BLCA, BRCA, HNSC, KIRC, LIHC, LUAD, LUSC, PRAD, THCA and UCEC. The distribution of the proportions was similar across the 10 cancer types, with HNSC showing the highest, and PRAD and THCA the lowest proportion of SOM-E variants. In all cancer types, the SOM-L variants represented a higher than SOM-E proportion of the total SOM variants. (**d**) Positive correlation (Spearman, *r*_*s*_) between VAF_tRNA_ and VAF_tDNA_ in the ten cancer types, and, in the pooled across-cancer data (**e**) Values of 0 and 1 on the y-axis represent SOM-L and SOM-E variants, respectively. (**f**) Distribution of variant allele frequency (V_R:D_) in the SOM-E, SOM, and SOM-L mutation categories.

The pipeline of our analysis is presented on Fig. 1a. From the originally selected for the study samples (Supplementary Table 1), we excluded those with insufficient purity assessments, extensive number of somatic mutations, and technical variables that can affect the assessments (See Methods). This retained 5523 high-confidence exonic SNVs in 3983 genes, 230 of which listed in the CGC (Supplementary Table 2). For each SNV, we quantitated the somatic allele frequency as the expressed variant allele fraction in the tumor RNA (VAF_tRNA_), including its extremes, where the somatic variant is either completely lost in the transcriptome (SOM-L, VAF_tRNA_ ~ 0) or over-expressed (SOM-E, VAF_tRNA_ ~ 1, Fig. 1b)^12^. Of note, SOM-L and SOM-E are called in the presence of a bi-allelic signal in the DNA (0 < VAF_tDNA_ < 1)^12^. The remaining somatic variants were designated as SOM. Across the ten cancer types, we called 350 SOM-E variants (6.3% of all variants) and 1044 (18.9%) as SOM-L (Fig. 1c and Supplementary Table 3). As compared to SOM-E, SOM-L SNVs were more frequent in all 10 cancers, with the difference ranging from 1.8-fold for HNSC, and 13-fold for PRAD and THCA. The highest proportion of SOM-E variants was observed in HNSC (consistent with another study exploring HNSC samples from TCGA^3^), LUSC, and BLCA. Next, we estimated the tumor expressed allele frequency (VAF_tRNA_) relative to the variant allele fraction in the tumor DNA (VAF_tDNA_), for which we used the previously introduced expression V_R:D_ = VAF_tRNA_/VAF_tDNA[1]_.

As expected, our analysis revealed positive correlation between VAF_tRNA_ and VAF_tDNA_ for the individual cancer types (Fig. 1d) and the pooled data across the ten cancer types (Spearman *r*_*s*_ = 0.38, p < 2.2e-16, Fig. 1e). The distribution of V_R:D_ in regards to the SOM-E, SOM and SOM-L categorization of somatic mutations is presented on Fig. 1f. In the presented below results, we used V_R:D_ to correlate with: (1) functional features of the mutation, including predicted effects on the protein, (2) position in genes that have been causally implicated in cancer, as defined by the CGC^17^, (3) total gene expression, and, (4) location in transcription factor binding sites (TFBS).

### Somatic allele expression in the context of the predicted function on the protein

**Figure 2:**
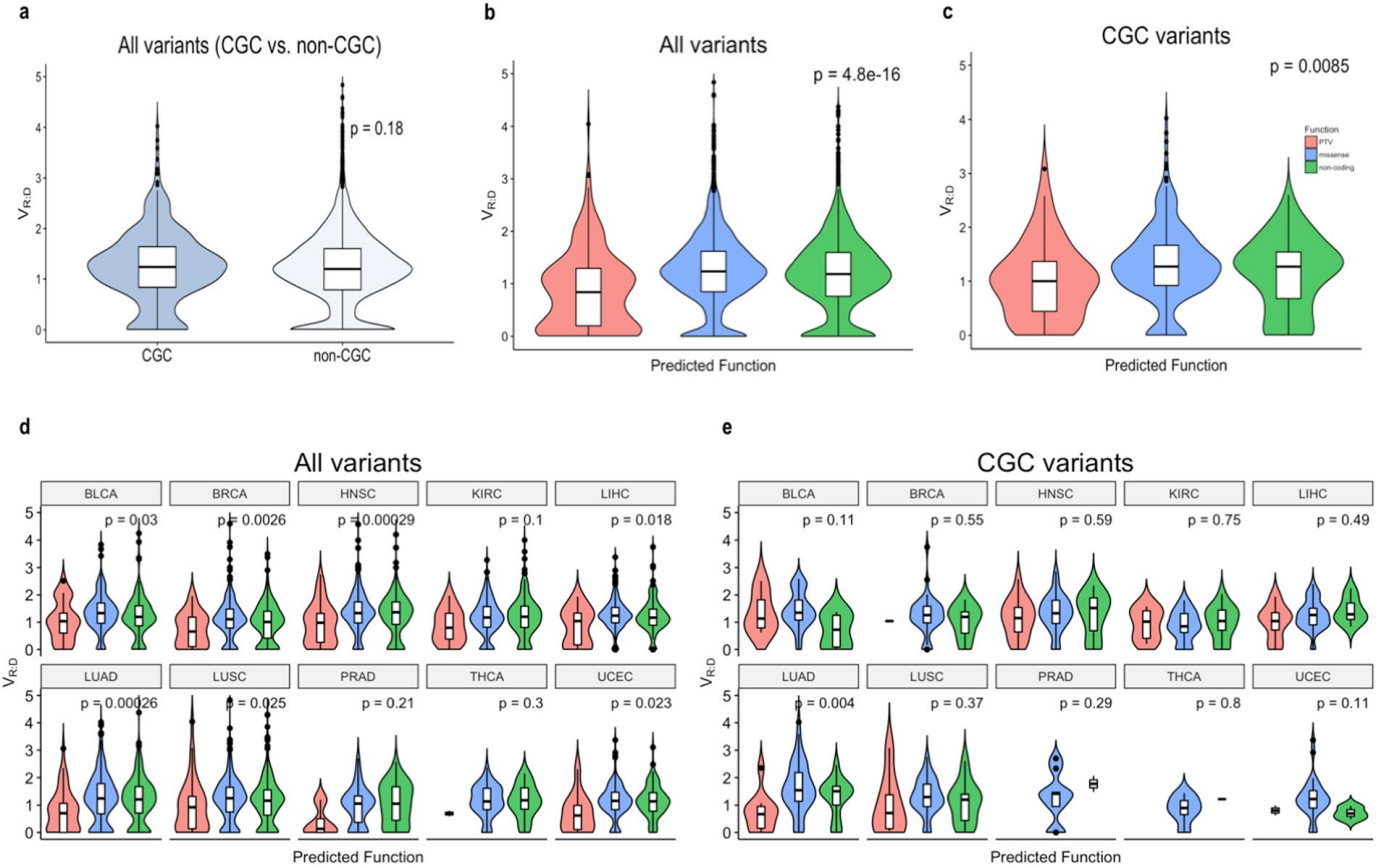
(**a**) Distribution of V_R:D_ in somatic mutations between CGC and non-CGC genes. (**b**) Distribution of V_R:D_ in somatic mutations categorized based on their predicted effect on the protein function in the entire dataset and in the CGC subset. (**c**) The mutation categories correspond to: generating a premature termination codon variant (PTV), substitution of an amino acid (missense) or not altering a coding sequence (silent). In both analyses the PTVs presented with significantly different distribution of V_R:D_ (p < 0.05) expressed as lower average allele fraction and a higher proportion of SOM-L mutations (V_R:D_ ~ 0). However, while still significantly different, the PTV V_R:D_ in the CGC genes appears more similar to the V_R:D_ of missense and silent variants, as compared to the pooled data from all genes. (**d)** Distribution of V_R:D_ in PTV, missense and silent variants in individual cancer types across the entire dataset, and (**e**) in CGC-genes.

**Figure 3:**
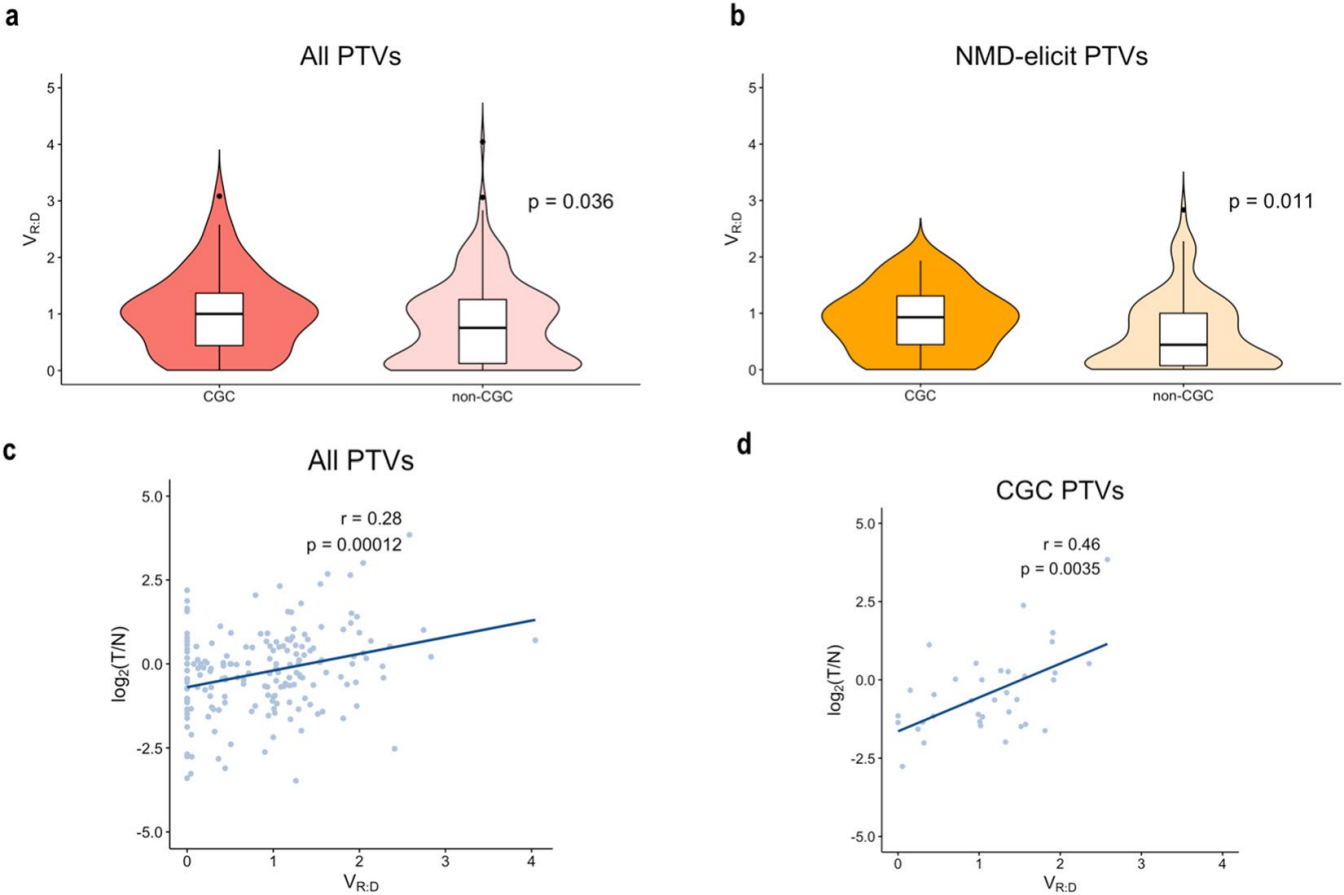
(**a)** Direct comparison of PTV V_R:D_ distribution  in CGC and non-CGC genes shows statistical significance (p = 0.036). The difference is more pronounced when exclusively NMD-elicit PTVs are analyzed (p = 0.011, **b**). (**c**) A positive correlation between V_R:D_ of PTVs and log2[T/N] (Spearman, *r*_*s*_) is seen for the PTVs in all genes and (**d**) in the CGC genes only.

We first assessed the distribution of V_R:D_ across the entire dataset, and compared variants located in CGC-genes, with those in the rest of the genome. As seen in Fig. 2a, the V_R:D_ distribution between variants in CGC and non-CGC genes did not show a statistically significant difference (p = 0.18). We next assessed the distribution of the tumor V_R:D_ across the different categories of somatic mutations with regards to their predicted effect on the protein: stop-codon generating (premature terminating variants, PTV), missense, and silent. Naturally, PTVs showed distinct allele expression as compared to the missense and noncoding variants (Kruskal-Wallis p = 4.8e-16, Fig. 2b). The difference presented both as lower average V_R:D,_ and higher proportion of mutations that are not expressed at all (SOM-L). This distribution of the variant allele fraction is suggestive for higher degradation rate of PTV-containing transcripts through nonsense-mediated mRNA decay (NMD)^4^ and concurs with the expected pattern and the observations from related studies^1–3^. However, when we analyzed the variants in the CGC group separately, we observed a different shape of the PTV V_R:D_ (Fig. 2c), with higher average allele frequency, and a lower proportion of SOM-L variants (See Fig. 3 and the corresponding section of the results). These effects were also apparent in the individual cancer types, although in some cancers the number of PTVs in GCG genes was low (Fig. 2d,e). Overall, in the CGC genes, the V_R:D_ distribution for PTVs was more similar to the V_R:D_ distributions of missense and silent variants.

### PTV-containing transcripts

To further explore the above-noted difference in the CGC PTV-alleles’ prevalence, we directly compared the V_R:D_ of PTVs located in CGC and in non-CGC genes. Consistent with the above, higher PTV V_R:D_ was seen in the CGC dataset (p = 0.036, Fig. 3a). Of note, a similar shape of the V_R:D_ distribution was seen in the individual cancer types with a number of PTVs in the CGC group sufficient for analysis (Supplementary Fig. 1a), where PTVs showed higher V_R:D_ and lower proportion of SOM-L variants in the CGC genes.

The observed higher allele frequency of somatic PTVs in CGC genes (as compared to the non-CGC genes) suggests a lower NMD-degradation rate of PTV-containing transcripts. To assess if this observation is due to a higher proportion of PTVs residing in NMD-escaping regions/genes in the CGC data set, we set out to perform our analysis exclusively on PTV-alleles predicted to be NMD-degraded (i.e. NMD-elicit PTVs), as defined by their position in the gene and the gene’s particular features. Accordingly, we removed from the analysis PTV-containing transcripts that are likely to escape NMD, combining escape rules defined by two major recent NMD studies^18,19^. Based on the findings in those studies, we filtered out mutations located: (1) more than 50 bp upstream of the last exon-exon junction, (2) in the first 200 nucleotides after the start codon where an alternative in-frame AUG is present, (3) in long exons, and positioned <250 nt from the closest exon boundary, (4) in short-living (<1 h half-life) transcripts^20,21^, (5) in single-exon genes, and, (6) in NMD-insensitive genes. This filtering retained 154 exclusively NMD-elicit PTVs (Supplementary Table 4), for which we re-estimated the variant allele frequency in CGC and non-CGC genes. Notably, in this stringent NMD-elicit group, the allele frequency distribution preserved its shape and the significance of the observed higher V_R:D_ in the CGC genes (p = 0.011, Fig. 3b). This result indicates that the high PTV allele expression in the CGC genes cannot be explained solely by NMD prediction based on mutation position, gene structure, and transcript processing dynamics. Instead, it suggests tolerance of cancer-implicated PTV-containing transcripts by the tumor transcriptional machinery.

We then set out to determine if the higher allele frequency of somatic PTVs in the tumor tissue correlates with total gene expression levels as compared to the matched normal tissue. To accomplish this, we estimated the expression changes of the genes bearing somatic mutations, measured as log_2_ of the fold-change between the expression levels in the tumor compared to the matched normal tissue (log_2_[T/N]) and correlated log_2_[T/N] with the variant allele frequency V_R:D_. Intriguingly, this analysis showed a positive correlation between V_R:D_ and the total expression change of PTV-bearing genes (*r*_*s*_ = 0.28, p = 1.2e-04, Fig. 3c), which further increased in the group of the CGC genes (*r*_*s*_ = 0.46, p = 3.5e-03, Fig. 3d).

**Figure 4:**
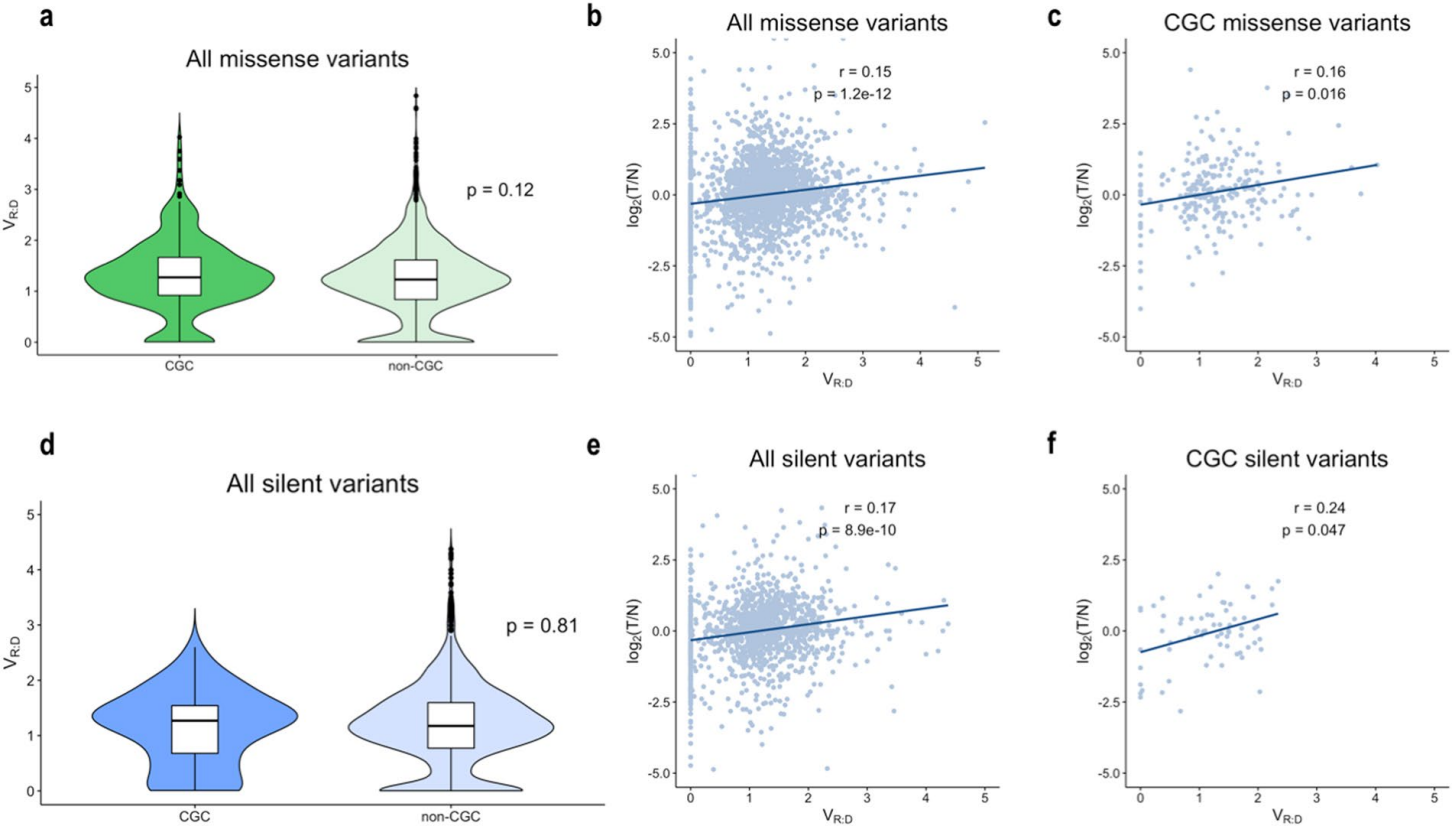
(**a**) V_R:D_ distribution for missense variants in CGC and non-CGC genes. (**b)** Correlation between V_R:D_ and expression change log2[T/N] (Spearman, *r*_*s*_), of missense variants in the entire dataset and in the CGC subset only (**c**). (**d**) V_R:D_ distribution for silent variants between CGC and non-CGC genes was similar (p = 0.81). (**e**) Correlation between V_R:D_ and log2[T/N] in the entire set of silent variants, and for those positioned in CGC genes only (**f**).

Next, we assessed the allele frequency in CGC and non-CGC genes for the missense and silent somatic variants in our dataset. The difference between CGC- and non-CGC-located missense and silent variants did not reach statistical significance (p = 0.12, and p = 0.81, respectively, Fig. 4); analogous patterns were seen in most of the analyses in the individual cancer types (Supplementary Fig. 1b,c, respectively).

### Correlation between allele-preferential and total gene expression in the entire dataset

**Figure 5:**
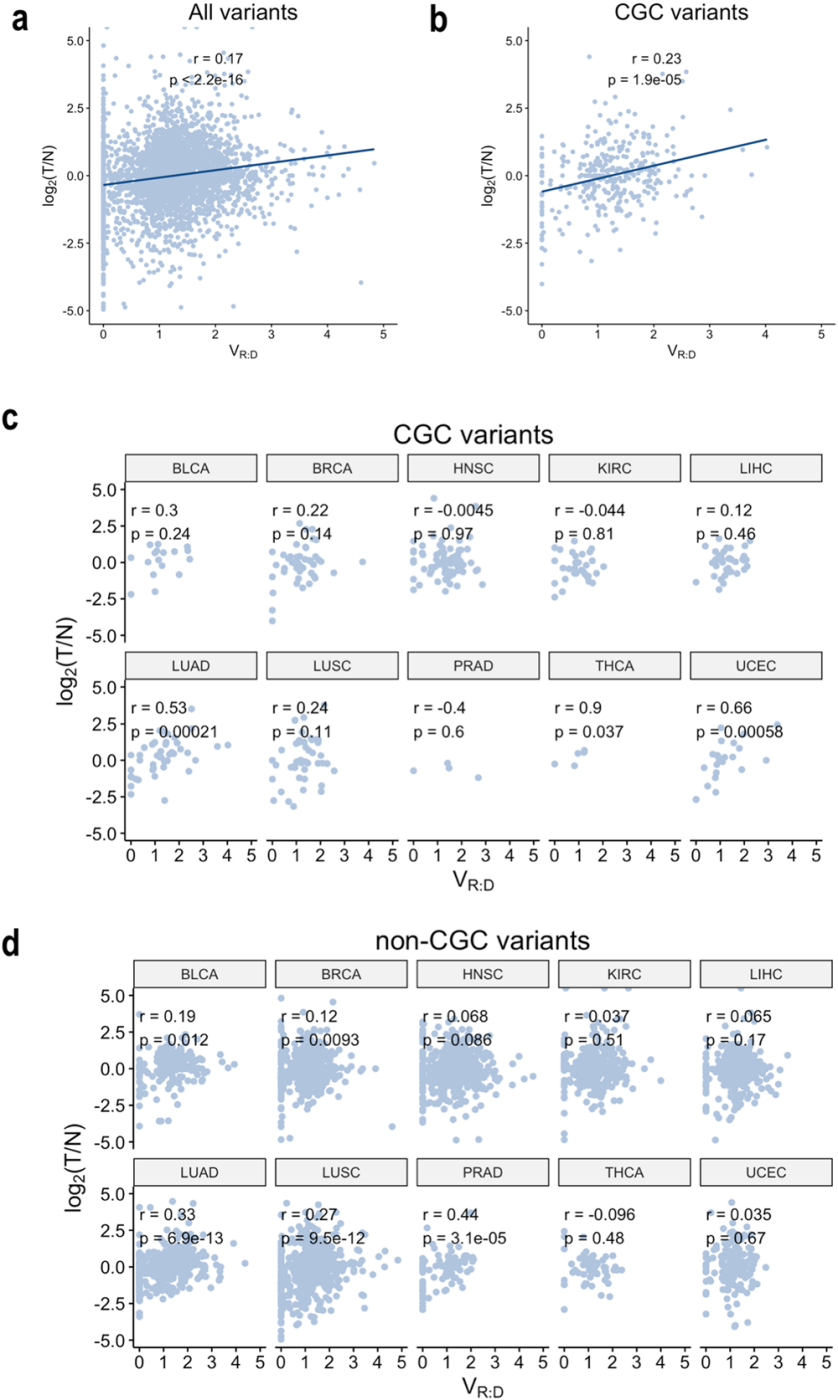
Correlation between log2[T/N] and V_R:D_ in the entire dataset (**a**) and the CGC genes subset (**b**). (**c**) Correlation between log2[T/N]) and V_R:D_ in the individual cancer types in CGC genes, and, non-CGC genes. (**d**) In the group of the CGC genes, LUAD, THCA and UCEC showed statistically significant correlations.

We then set out to determine if there is a correlation between allele frequency and total gene expression of the variant-harboring gene relative to the matched normal tissue. This analysis revealed a positive correlation between V_R:D_ and total gene expression change across the entire dataset (*r*_*s*_ = 0.17, p < 2.2e-16, for the pooled variants, Fig. 5a), as well as in the CGC subset (*r*_*s*_ = 0.23, p = 1.9e-05, for the pooled variants, Fig. 5b). In three of the individual cancer types, LUAD, THCA and UCEC, we observed a significant correlation between V_R:D_ and log_2_[T/N] in the CGC gene-set. In these three cancer types, the observed in the CGC gene-set correlation was substantially stronger as compared to the one seen in the entire gene-set (LUAD, *r*_*s*_ = 0.53, p = 2.1e-04, THCA: *r*_*s*_ = 0.9, p = 0.037, and UCEC: *r*_*s*_ = 0.66, p = 5.8e-4 (Fig. 5c,d).

We then outlined the extreme subsets of somatic variants with co-occurring high expressed allele frequency and increased or decreased total expression of the harboring gene. To do that, we selected variants with V_R:D_ > 2, (indicating at least 2-fold higher expressed allele frequency (VAF_tRNA_), as compared to the DNA allele frequency (VAF_tDNA_)), and at least a two-fold increase or decrease in the total gene expression in the tumor as compared to the normal tissue. A total of 107 somatic variants had expressed allele frequency co-occurring with increased gene expression. In another 73, the increased allele frequency co-occurred with a more than 2-fold decrease in the total expression of the harboring gene (Supplementary Tables 5 and 6).

### Correlation of allele frequency and predicted functionality

We then tested V_R:D_ in correlation with mutation pathogenicity as modeled through several popular functionality predictive tools: Polyphen (PPH)^22^, Combined Annotation Dependent Depletion (CADD)^23^, Genomic Evolutionary Rate Profiling (GERP)^24–26^, and Functional Analysis Through Hidden Markov Models (FATHMM)^27,28^. We did not observe a strong correlation between the V_R:D_ and the pathogenicity value computed by FATHMM (Supplementary Fig. 2a,b). However, when we assessed the categorical FATHMM estimations (categorized as either “pathogenic” or “neutral”), higher V_R:D_ was seen in the pathogenic variants (p = 0.023, Supplementary Fig. 2c). No strong correlations between V_R:D_ and CADD, GERP or PolyPhen scores of the variants in the CGC genes and non-CGC genes were observed (Supplementary Fig. 2d–i).

### Somatic allele-frequency and TFBS

**Figure 6:**
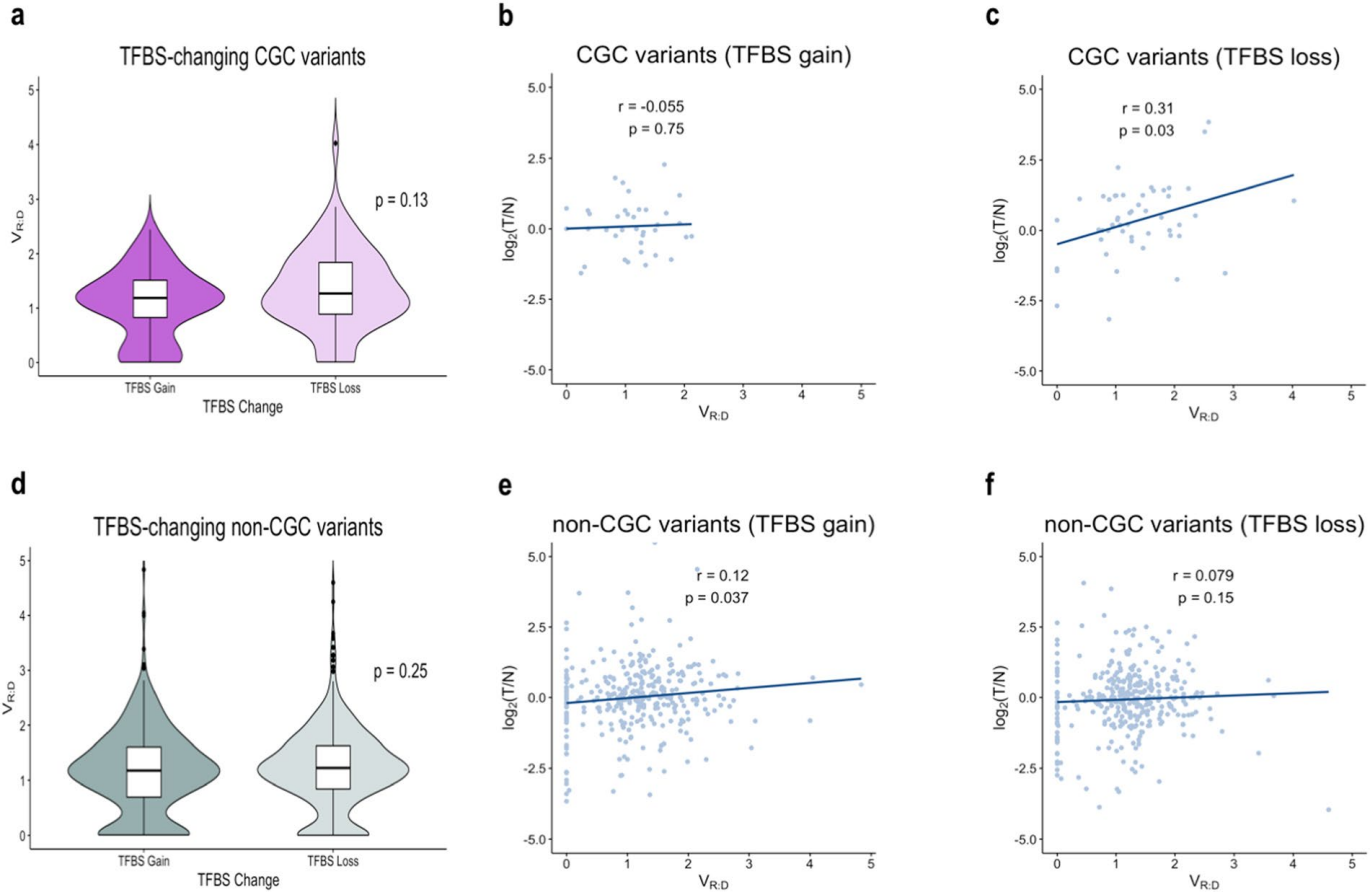
(**a**) V_R__:D_ distribution for somatic variants that generate a new TFBS (TFBS-gain) as compared to those that disturb an existing TFBS (TFBS-loss). (**b**) Correlation between log2[T/N] and V_R:D_ TFBS-gain variants, and in TFBS-loss variants (**c**). (**d)** Distribution of V_R:D_ between TFBS-gain and TFBS-loss somatic variants in non-CGC genes; no difference was observed. (**e)** Correlation between log2[T/N] and V_R:D_ in TFBS-gain variants in the CGC genes, and in the non-CGC genes (**f**).

We next examined if somatic allele frequency correlates with residence of the mutation in motifs recognizable by transcription factors (i.e transcription factor binding sites, TFBS). To accomplish this, we assessed the variant and the reference motifs for TFBS using TRANSFAC^29^, and then categorized the variants in the following three groups: generating a new TFBS (TFBS-gain, 2736 variants), destroying an existing TFBS (TFBS-loss, 1980 variants), and not changing a known motif (Supplementary Table 7). We then compared the allele-frequency and the total gene expression among the three categories. We did not observe a statistical difference between TFBS-gain and TFBS-loss variants in the CGC-subset (p = 0.13, Fig. 6a), however, in the TFBS-loss variants we detected a positive correlation with the gene expression levels (***r***_***s***_ = 0.31, p = 0.03). This observation is consistent with degradation (by the somatic mutations) of existing motifs for transcription suppressing factors (TFs). Because most of the TFs found to recognize somatically altered motifs are known to act both as activators and suppressors of the transcription, it was not possible to impartially determine if suppressing or activating activity is associated with allelic and total expression (See Supplementary Table 7).

### Purity and CNA assessments

V_R:D_ reflects both admixture of the tumor sample with non-tumor material and CNAs. To further delineate the effects of purity and CNAs on the observations, we obtained: (1) purity of each sample, as estimated by 5 different methods: Estimate, Absolute, LUMP, IHC, and the Consensus Purity Estimation (CPE)^13^, and, (2) the CNAs assessments measured as genomic segment mean values (log2(copy-number/2)) from TCGA.

Because purity affects both DNA and RNA content, we directly weighted the relative (to DNA) expressed allele frequency - V_R:D_ - by the proportion of the sample estimated by each of the above methods to correspond to tumor tissue and used these adjusted values to perform the analyses described above. The results are presented in Supplementary Figs 3–7. Overall, most of the observations retained their significance. Notably, ESTIMATE- and IHC-weighted V_R:D_ (eV_R:D_ and iV_R:D_, respectively) produced a higher number of significant observations as compared to the unweighted V_R:D_ (Supplementary Figs 3 and 4). For example, the difference in eV_R:D_ between CGC and non-CGC variants across the entire dataset reached statistical significance (p = 0.05, See Supplementary Fig. 3b).

To assess if CNAs contribute to the observations and more specifically to the differences between SNVs in CGC and non-CGC genes, we compared the absolute segment means between CGC and non-CGC genes in the context of their harbored SOM-E and SOM-L variants (Supplementary Fig. 8). This analysis showed similar distributions of the absolute segment means between variants in CGC and non-CGC genes in the entire dataset (Supplementary Fig. 8a), in the groups of the SOM-E and SOM-L variants (Supplementary Fig. 8b,c, respectively), as well as in the groups of SOM-E PTVs and SOM-L PTVs (Supplementary Fig. 8d,e, respectively). These observations suggest that in the studied dataset, CNAs are unlikely to substantially contribute to the observed differences between CGC and non-CGC genes. Aligned with the above, the positive and negative segment means (corresponding, respectively, to amplifications and deletions) did not correlate with SOM-E or SOM-L estimations (Supplementary Fig. 9a,b).

We note that several factors that can affect the presented observations remain unaccounted for in our study. Technical variables related to differences in the RNA (and DNA) extraction, sequencing library generation and processing, depth of sequencing, and sequencing platform can affect the presented observations, including the proportion of transcripts containing PTVs. Importantly, if RNA and DNA are not extracted from the same tumor specimen, they may have different features, including purity and CNAs. Furthermore, “normal” tissue obtained from a tumor-adjacent site may confer expression patterns affected by exposure to tumor signals. While TCGA represents a major effort for uniformity of tumor collection and processing, it is important to consider possible effects of the above factors on the expressed variant allele frequencies. Finally, the threshold for minimal read counts per loci selected for analyses can impact the computations. Naturally, low thresholds include a higher number of variants but decrease the confidence of VAF estimations for under-expressed genes, while high thresholds support confident VAF measurements in a lower total number of variants and exclude genes with low expression. Our analyses with a range of different thresholds on subsets of the herein analyzed datasets showed comparative outcomes^12^. However, the effects of the read count threshold on the VAF assessments are important to contemplate.

### Genes with multiple somatic variants

**Figure 7:**
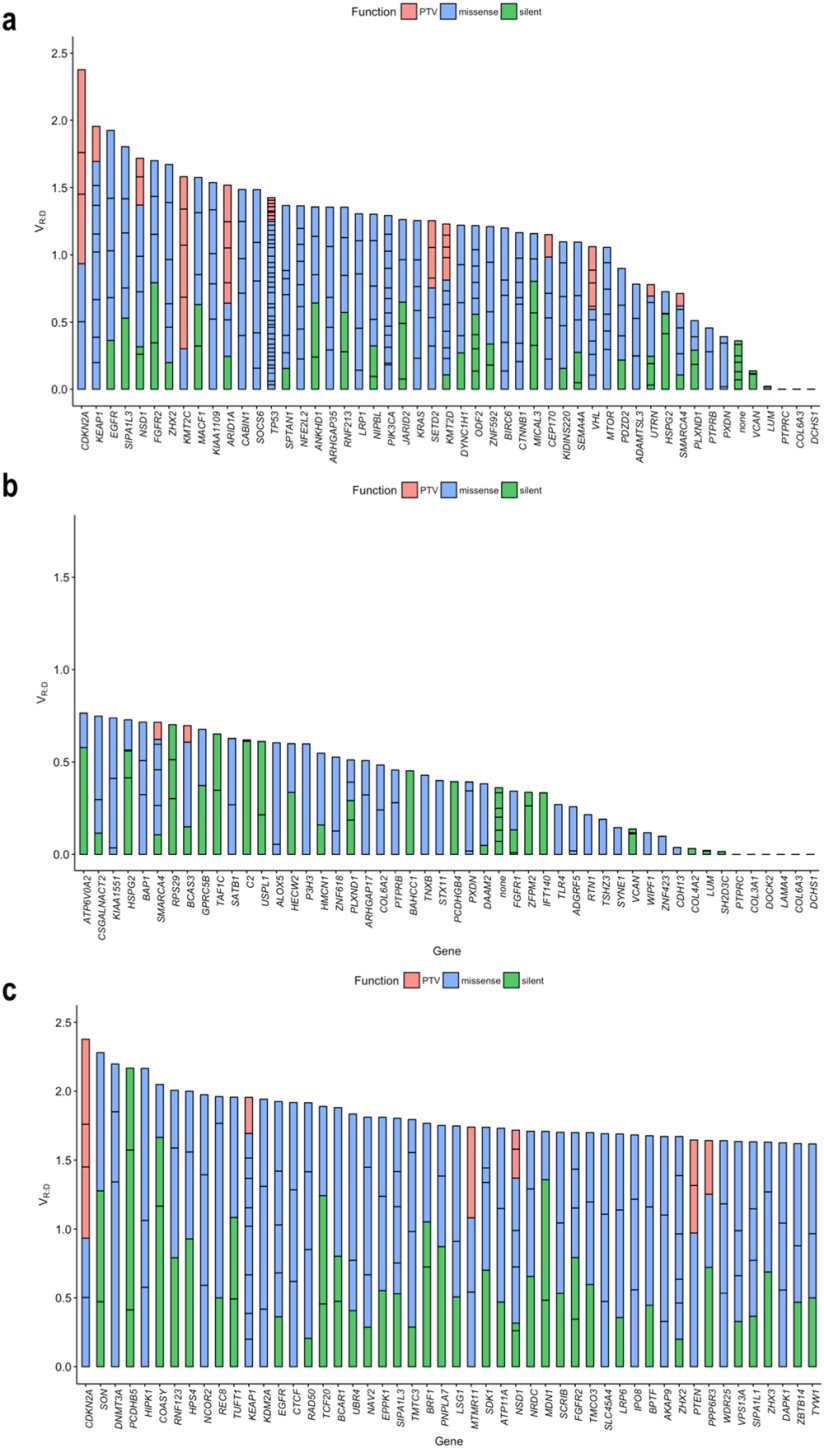
(**a**) Genes with more than 5 somatic variants (in the entire dataset) ranked by mean V_R:D_. (**b**) The set of 50 genes with more than 3 somatic variants with lowest mean V_R:D_. (**c**) Top 50 genes with more than 3 somatic variants with the highest V_R:D_; the gene-set is enriched in CGC genes and includes *CTCF*, *CDKN2A*, *CDK12*, *DNMT3A*, *PTEN*, *KMT2C*, and *KEAP1*.

In our dataset, 49 genes had 5 or more somatic mutations (Fig. 7a and Supplementary Table 8). To examine the V_R:D_ in genes with multiple somatic mutations, we ranked the genes by their mean V_R:D_.

Naturally, owing to the high somatic mutation frequency used for the selection, this gene-set was enriched in CGC genes (p < 0.001, chi-square test). From this gene-set, three NMD-sensitive genes had multiple NMD-elicit PTVs with V_R:D_ values similar or higher to the V_R:D_ values of missense and silent mutations in the same gene: *ARID1*, *TP53*, and *NSD1* (all of them CGC genes).

We then examined the set of 283 genes with 3 or more somatic mutations (Supplementary Table 9). While this dataset was also significantly enriched in CGC genes, no over-representation of CGC genes was seen in the 50 genes with the lowest mean V_R:D_ (Fig. 7b). The CGC genes with lowest mean V_R:D_ based on 3 and more mutations were the protein tyrosine phosphatases *PTPRB* and *PTPRC*, and *FGFR1*. Naturally, low V_R:D_ confers features that imply intolerance by the transcriptional machinery of the harbored variant. Apart from CNAs and NMD, possible factors contributing to the SOM-L prevalence are (1) low DNA allele frequency and related technical variabilities and (2) general infidelity of the cancer transcriptional machinery. Additional factors, such as estrogen receptor (ER) expression levels, are also reported to correlate with the number of expressed somatic mutations in breast cancer^2^. All these factors are likely to dilute functional annotations in the SOM-L mutations. In contrast, the set of the top 50 genes with high mean V_R:D_ based on 3 or more somatic variants was significantly enriched with CGC genes (p < 0.01, Fig. 7c), including *CTCF*, *CDKN2A*, *CDK12*, *DNMT3A*, *PTEN*, *KMT2C*, and *KEAP1*^30^.

## Discussion

Despite the growing accessibility of human cancer sequencing data, the number of available normal and tumor DNA and RNA datasets from the same individual/sample is still limited. In contrast to tumor expression data alone, the matched normal transcriptome provides the opportunity to directly assess normal-to-tumor expression changes, and to link them to features of interest in the tumor sample, including somatic variants’ allele frequency and functionality. In this study, we employed the unique set of matched tumor and normal RNA and DNA sequencing data obtained from TCGA to assess somatic allele frequency, analyze the correlation between allele expression and functionality of somatic SNVs, and connect to potentially mutation-instigated expression changes. Ultimately, the major outcome from our study is the demonstration of the vast value of the information provided by somatic allele frequency. In addition, several striking observations were apparent from our analyses.

First, somatic variants in CGC genes were more frequently expressed and had higher allele frequency in comparison to the somatic variants in the rest of the genes. Second, variants with high predicted functionality and/or pathogenicity generally had increased allele frequency as compared to neutral variants, and this observation was stronger in the subset of the CGC genes. High somatic allele frequency co-occurring with increased gene expression level is consistent with variant-mediated transcriptional up-regulation, acting in cis-fashion^6^. Alternatively, the mutant transcripts could be favored by the tumor cell due to a growth or a survival advantage provided by the mutation. Both scenarios infer functionality and/or tumorigenic potential, which are frequent features of mutations in tumor suppressors and oncogenes, and hence concur with the observed allele behavior in the CGC subset. Comparatively, high somatic allele frequency co-occurring with decreased total expression of the gene can indicate more complex scenarios where the variant allele is selected in the tumor transcriptome possibly due to down-regulation of the produced protein. The variants outlined by our analysis represent examples that can be further explored for consistency with the herein proposed mechanisms.

The observations above are largely consistent across the ten individual cancer types, as well as with our previous findings on breast cancer, and related studies by others^1–3^. All these studies illustrate the general tendency of the cancer transcriptome to favor advantage supplying variants, which are ultimately those with higher functionality and pathogenic potential^1^. The new layer provided by this study is the linking of gene expression changes in the tumor as compared to the matched normal tissue, which further affirms the previous observations. Most importantly, it represents a step towards the interpretation of the downstream effects of the somatic variants. In most of our analysis, expression changes correlated positively with somatic allele frequency. Naturally, this is not surprising as the absolute allele quantity contributes both to the allele frequency and the total gene expression. However, an unexpected notable finding from the expression analysis is the high expression of the PTV-bearing transcripts in the CGC genes, which suggests that these genes are more frequently targets of NMD-escaping somatic variants.

Several explanations might account for the higher frequency of NMD-escaping PTVs in the CGC genes. Because CGC is a diverse group containing both oncogenes and tumor suppressor genes, it is reasonable to presume that the observation results at least partially from non-specific effects. For one, it is possible that PTV-transcripts and their encoded shorter proteins non-specifically (i.e. through depleting the cellular molecular sources) prevent the normal cellular processes from counteracting the tumorigenic activity, and are thus favored as advantage-supplying features. In line with the above, it is also possible that the dynamic growth and replication of the tumor cell does not allow enough time for the NMD machinery to allocate molecular resources and properly degrade newly arisen (somatic) PTV-containing transcripts. The above effects would be more pronounced in the CGC subset if the CGC genes were more actively transcribed (and degraded) in the tumor cell. The latter is an attractive possible explanation, especially given that short-living transcripts more frequently escape NMD^19^. Thus, by a wider definition of oncogenic action, the NMD-escaping transcripts might act as advantage-supplying molecules via general dysregulation of cellular functioning. It is not possible to predict from our analyses whether these PTV-containing transcripts are translated into shorter proteins. Nevertheless, NMD-escaping mutations appear to target CGC genes at a higher frequency than the rest of the genome, and this observation demands further studies, including at the protein level.

While generally aligned with previous studies, our results highlight several previously unnoted observations; therefore, we have attempted to analyze possible explanations. First, our expression analysis is based on comparisons with matched normal tissue. In contrast, most of the large analyses opt not to confine their studies exclusively to matched normal-tumor expression datasets, mainly because of the low number of samples with both normal and tumor transcriptome sequencing data^16,18,19^. Instead, many studies use mean and median expression estimates^18,19^, which, albeit stringently corrected for sample subtypes including expression patterns, represent approximations of the actual expression in the individual sample. In contrast, we estimate the exact expression changes between the normal and the tumor transcriptome and compensate for the low sample count with an across-cancer analysis. Indeed, the most significant observations hold true in the individual cancer types or are codirectional to the pooled dataset, which increases the confidence of our observations.

Furthermore, technical adjustments are also likely to be reflected in the outcome of our analyses. In regards specifically to the NMD, our analysis did not include variants with splice annotations. Many of the splice SNVs are predicted to result in NMD and are therefore frequently considered by NMD studies^18,19^. We choose not to include splice annotations because many splice-variants (especially those located in exons) are shown to lead to alternative splicing only in a proportion of the bearing molecules^31,32^, and systematic quantitation of this proportion is presently unavailable. By design, our study is confined strictly to exonic variants, where the comparative assessment of DNA and RNA allele frequency is minimally affected by technical variables. Another factor, this one impacting the entire dataset, is that we utilize the latest release of the human reference genome – hg38, using the high confidence curated alignments and variant calls directly from TCGA^16^. While hg38 arguably supplies higher annotation accuracy, it is still in the process of assimilation into the genome-studying research community, and most of the major NMD studies so far have used hg19^18,19^, which can partially account for the differences between these studies and our own.

In summary, we believe that our analyses present novel and intriguing insights into the allele-preferential expression of somatic mutations in cancer. Importantly, ranking of genes based on somatic allele frequency scores key cancer genes at top positions, and thus suggests that high somatic allele frequency can be used to indicate potential carcinogenic variants, and possibly, to identify cancer-driving genes.

## Methods

### TCGA samples

To systematically quantify somatic allele prevalence, we compiled data on single nucleotide somatic variants from The Cancer Genome Atlas (TCGA) for patients for whom the following sequencing datasets were available: normal exome (Nex), normal transcriptome (Ntr), tumor exome (Tex), and tumor transcriptome (Ttr). We selected 12 cancer types: BLCA, BRCA, COAD (Colon Adenocarcinoma), HNSC, KIRC, LIHC, LUAD, LUSC, PRAD, STAD (Stomach Adenocarcinoma), THCA, and UCEC, that had more than 10 samples with all four sequencing datasets. In addition, we required each sample to have at least three of the following five purity estimates - Estimate, Absolute, LUMP, IHC, and the consensus purity estimate (CPE), (See Supplementary Table 1), as well as CNA estimation (genomic segment means based on Genome-Wide-SNPv6 hybridization array)^13,33–35^. This initial set consisted of 416 individuals. From those, we excluded samples with an extensive number of somatic mutations (more than 1.5 interquartile ranges (IQR) above the third quartile, in our dataset 56), possibly due to clustered genomic rearrangements or other rare mechanisms of acquirement of somatic mutations^36,37^. As a result, COAD and STAD sets retained less than 10 patients suitable for assessment and were removed from further analyses. On the remaining samples, we applied stringent filters to ensure compatible RNA and DNA frequency estimation and to minimize effects due to CNAs, technical variables, and admixture with non-tumor cells. Importantly, we filtered out variants residing in imprinted genes (See Methods)^14,15,38,39^. These filtering steps yielded 5523 high-confidence exonic SNVs in 3983 genes, from which 230 were listed in the CGC (See Supplementary Table 2).

### Allele count and expression level computation

All the datasets were generated through paired-end sequencing on an Illumina HiSeq platform. The human genome reference (hg38)-aligned sequencing reads (Binary Alignment Maps, .bams) and the Simple Nucleotide Variation mutation annotation file (SNV.maf) were downloaded from the Genomic Data Commons Data Portal (https://portal.gdc.cancer.gov/) and processed downstream through an in-house pipeline. The RNA and DNA alignments, together with the variant lists were processed through RNA2DNAlign^12^. RNA2DNAlign produced variant and reference sequencing reads counts for all the variant positions in all four datasets (normal exome, normal transcriptome, tumor exome and tumor transcriptome). Selected read count assessments were visually examined using Integrative Genomics Viewer^40^. We excluded from further analyses variants which (1) were covered with less than 10 sequencing reads in the tumor DNA or the RNA sequencing data; (2) reside in known imprinted regions, and (3) were present in the normal DNA or RNA, suggesting germline origin. Variants positioned in the X Chromosome and on stably imprinted autosomal genes^14,15^ were excluded from the analyses. For the NMD-analysis, short-living (<1 h half-life) transcripts were identified based on Tani *et al*.^20,21^. The gene expression was quantified using the Cufflinks package from the Tuxedo suite, as we have previously described^41^.

### Assessment for allele distribution

Allele frequencies within a sample were determined through estimation of the relative abundance of variant over total sequence read counts, expressed as Variant Allele Fraction (VAF). For each somatic mutation, we computed the VAF = n_var_ /(n_ref_ + n_var_), for both tumor RNA (VAF_tRNA_) and tumor DNA (VAF_tDNA_), where n_ref_ and n_var_ are the counts of the reference and variant sequencing reads covering the position, respectively. To account for allele asymmetries related to DNA, we analyzed VAF_tRNA_ in the context of the corresponding VAF_tDNA_. Over-expression of somatic mutations (SOM-E status) was defined as prevalence of variant sequencing reads in the transcriptome (VAF_tRNA_ ~ 1), while SOM-L was defined by complete loss of the mutant allele in the transcriptome (VAF_tRNA_ ∼ 0)^12^. VAF_tRNA_ relative to VAF_tDNA_ (V_R:D_) was computed as previously described: V_R:D_ = VAF_tRNA_/VAF_tDNA_. Weighting for purity was performed by multiplying V_R:D_ by the proportion of the sample assessed to correspond to the tumor tissue component (i.e. aV_R:D_ = V_R:D_xABSOLUTE, iV_R:D_ = V_R:D_xIHC, eV_R:D_ = V_R:D_xESTIMATE, lV_R:D_ = V_R:D_xLUMP, and cV_R:D_ = V_R:D_xCPE).

### Functional and enrichment analyses

Functional annotations and conservation scores were extracted using the SeattleSeq annotation 147 (http://snp.gs.washington.edu/SeattleSeqAnnotation147/index.jsp). Pathogenicity was modeled using PolyPhen, CADD and FATHMM methods, and conservation was assessed based on GERP scores^23–28^. Transcription factor binding sites were analyzed using TRANSFAC 7.0^29^.

### Statistics

SOM, SOM-E and SOM-L variants were called based on a binomial test for variant and reference sequencing read distribution, as previously described^12^. The distributions of the variant allele frequency was assessed using Kruskal-Wallis rank sum test, and the Spearman rank correlation coefficient^42^. P-values below 0.05 were considered significant.

### Availability of data and materials

The datasets supporting the conclusions of this article are included within the article and its additional files.

## Electronic supplementary material

Supplementary Figures

Supplementary Tables
