# Supplementary material for: Systematic pan-cancer analysis of somatic allele frequency
Source: Sci Rep. 2018 May 16;8:7735. doi: 10.1038/s41598-018-25462-0 (PMC5956099; doi:10.1038/s41598-018-25462-0)
Supplement: Supplementary file 1 — Supplementary Figures [file 41598_2018_25462_MOESM1_ESM.pdf]

Systematic pan-cancer analysis of somatic allele frequency

Liam Spurr[1,2], Muzi Li[2,3], Nawaf Alomran[2,3], Qianqian Zhang[1,4], Paula Restrepo[1,2], Mercedeh Movassagh[2,5], Chris Trenkov[2], Nerissa Tunnessen[2], Tatiyana Apanasovich[6], Keith A. Crandall[7], Nathan Edwards[2,3], Anelia Horvath[1,2,4,7*]

[1]Department of Pharmacology and Physiology, School of Medicine and Health Sciences, The George Washington University, Washington, DC 20037, USA

[2]McCormick Genomics and Proteomics Center, School of Medicine and Health Sciences, The George Washington University, Washington, DC 20037, USA

[3]Department of Biochemistry and Molecular and Cellular Biology, Georgetown University, School of Medicine, Washington, DC 20057, USA

[4]Department of Biochemistry and Molecular Medicine, School of Medicine and Health Sciences, The George Washington University, Washington, DC 20037, USA

[5]University of Massachusetts Medical School, Program in Bioinformatics and Integrative Biology, Worcester, MA

01605, USA

[6]Department of Statistics, The George Washington University, Washington, DC 20037, US

[7]Computational Biology Institute, Milken Institute School of Public Health, The George Washington University, Washington, DC, 20052, USA.

**Correspondence to horvatha@gwu.edu*

Supplementary Figure 1. Distribution of $V_{R:D}$ in somatic mutations categories in CGC vs. non-CGC genes based on their predicted effect on the protein function: (**a**) Premature terminating variants, PTVs, (**b**) Missense variants (**c**) Non-coding variants. In the majority of the comparisons, higher $V_{R:D}$ was estimated in the CGC genes.

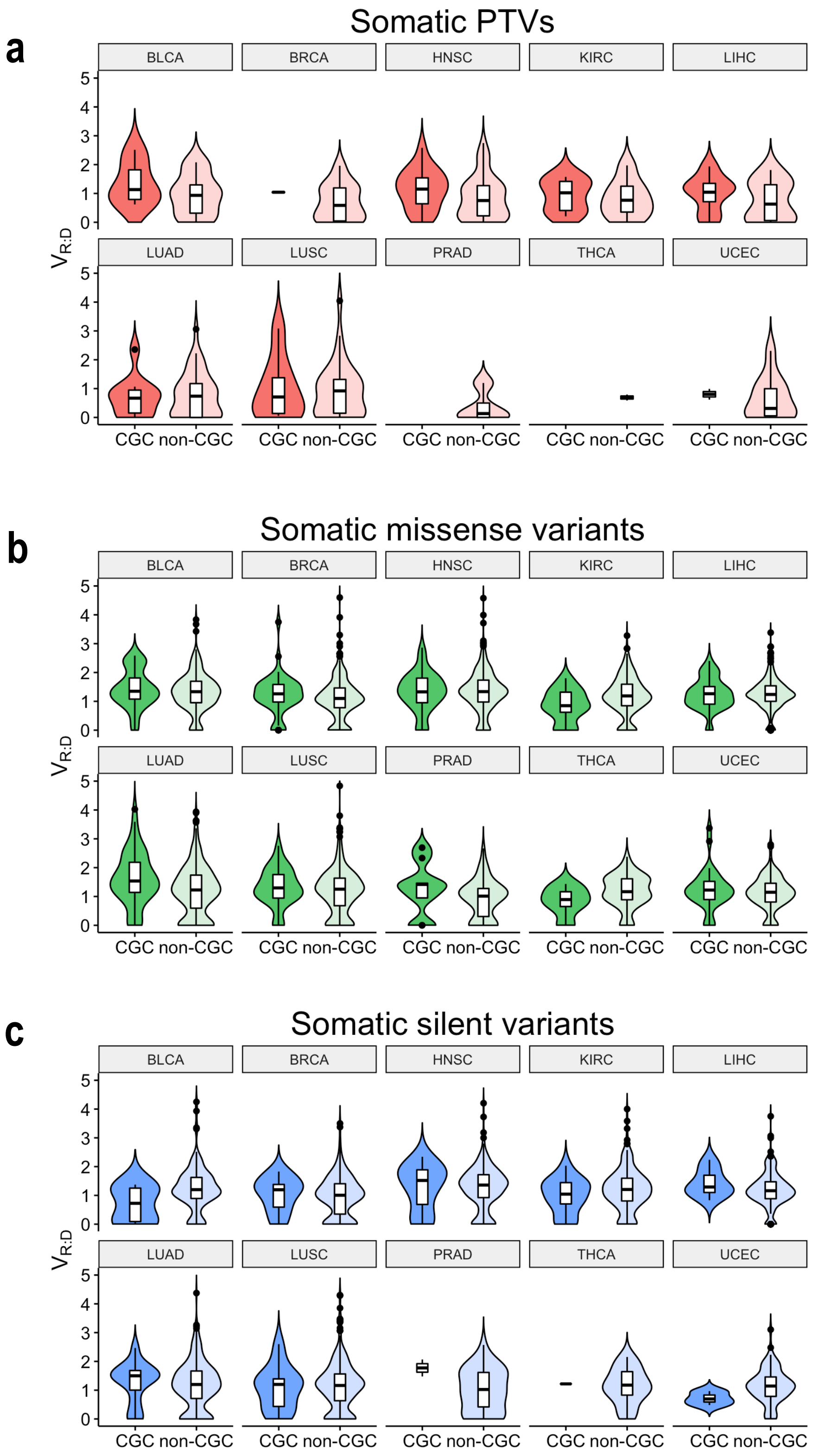

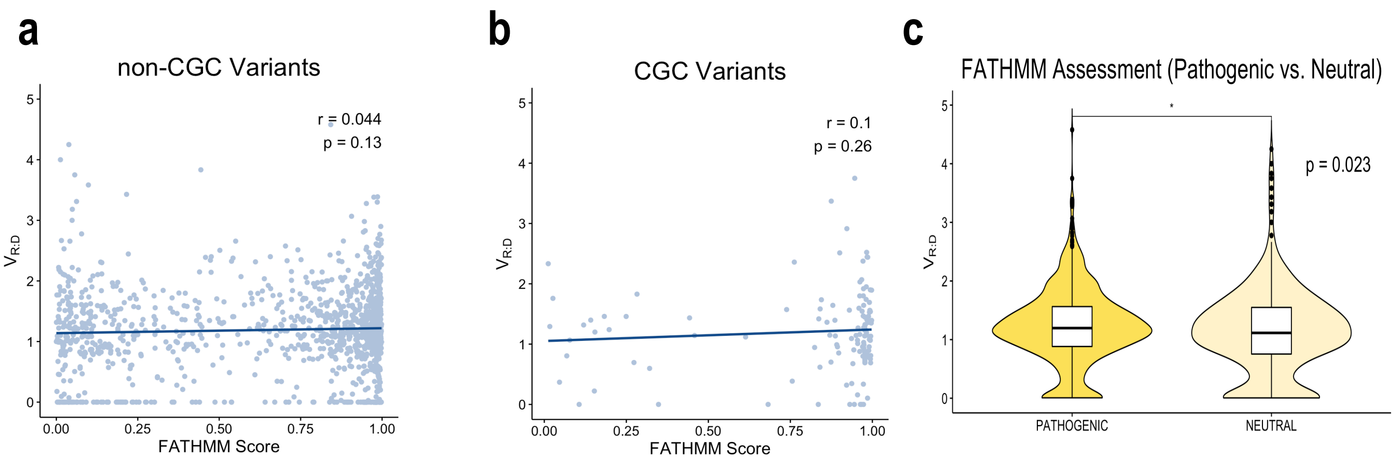

Supplementary Figure 2. a. Correlation between $V_{R:D}$ and pathogenicity score predicted through FATHMM for non-CGC variants, and (**b**) for CGC variants. **c.** Distribution of $V_{R:D}$ in pathogenic vs. neutral somatic variants as assessed by FATHMM. **d.** Correlation between $V_{R:D}$ and PolyPhen score for non-CGC variants, and (**e**) for CGC variants. **f.** Correlation between $V_{R:D}$ and GERP score for non-CGC variants, and (**g**) for CGC variants. **h.** Correlation between $V_{R:D}$ and GERP score for non-CGC variants, and (**i**) for CGC variants.

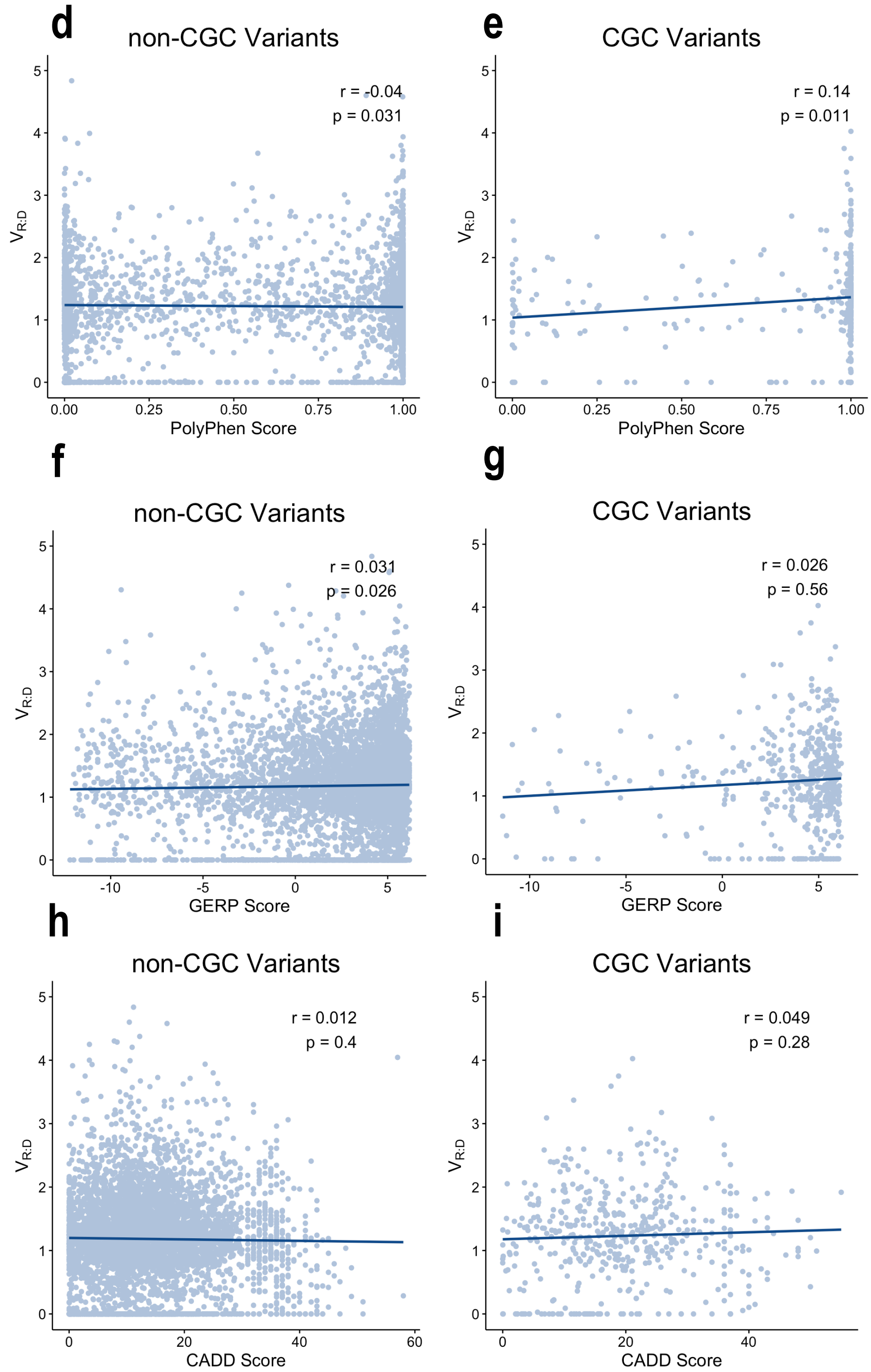

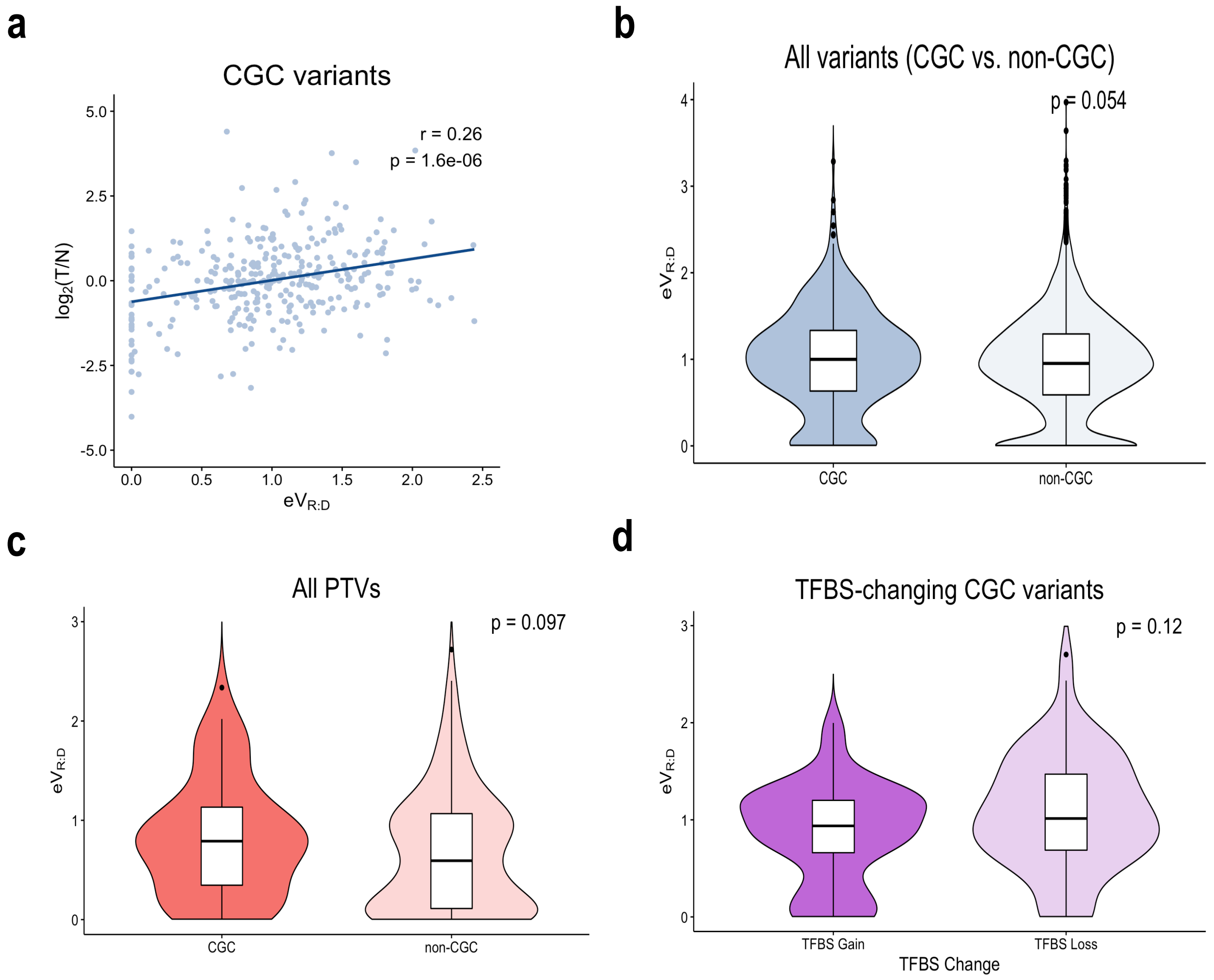

Supplementary Figure 3. Analyses of variant allele frequency adjusted through ESTIMATE-assessed purity ($eV_{R:D}$). **a.** Correlation between variant allele fraction and gene expression change. **b**. Distribution of variant allele frequency of CGC- and non-CGC somatic variants . **c**. Distribution of variant allele frequency in PTVs in CGC and those in non-CGC. **d**. Distribution of variant allele frequency in somatic variants that generate a new TFBS and those that destroy an existing TFBS. The results are co-directional with the other purity adjusted estimations.

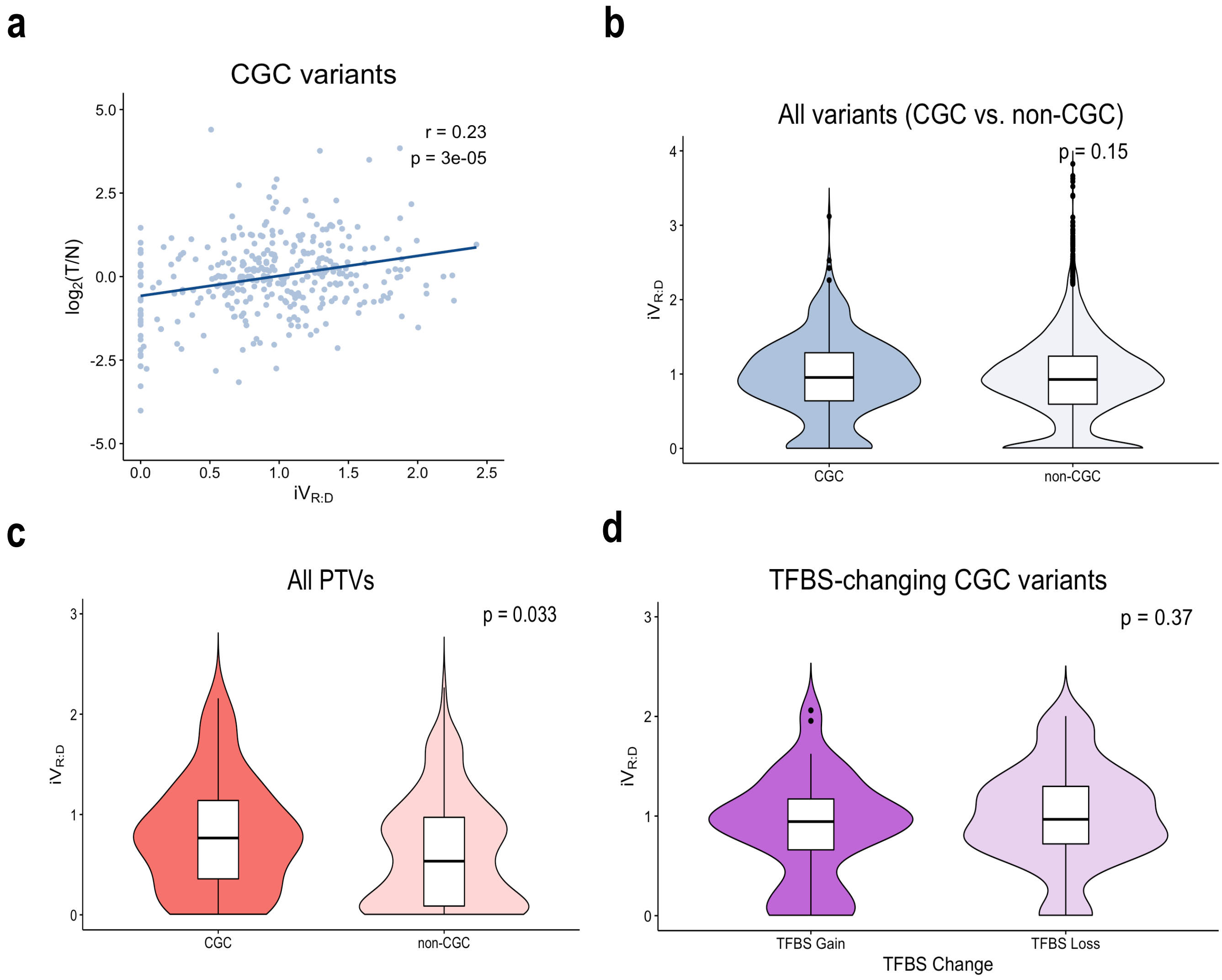

Supplementary Figure 4. Analyses of variant allele frequency adjusted through IHC-assessed purity (iVR:D). **a.** Correlation between variant allele fraction and gene expression change. **b**. Distribution of variant allele frequency of CGC- and non-CGC somatic variants . **c**. Distribution of variant allele frequency in PTVs in CGC and those in non-CGC. **d**. Distribution of variant allele frequency in somatic variants that generate a new TFBS and those that destroy an existing TFBS. The results are co-directional with the other purity adjusted estimations.

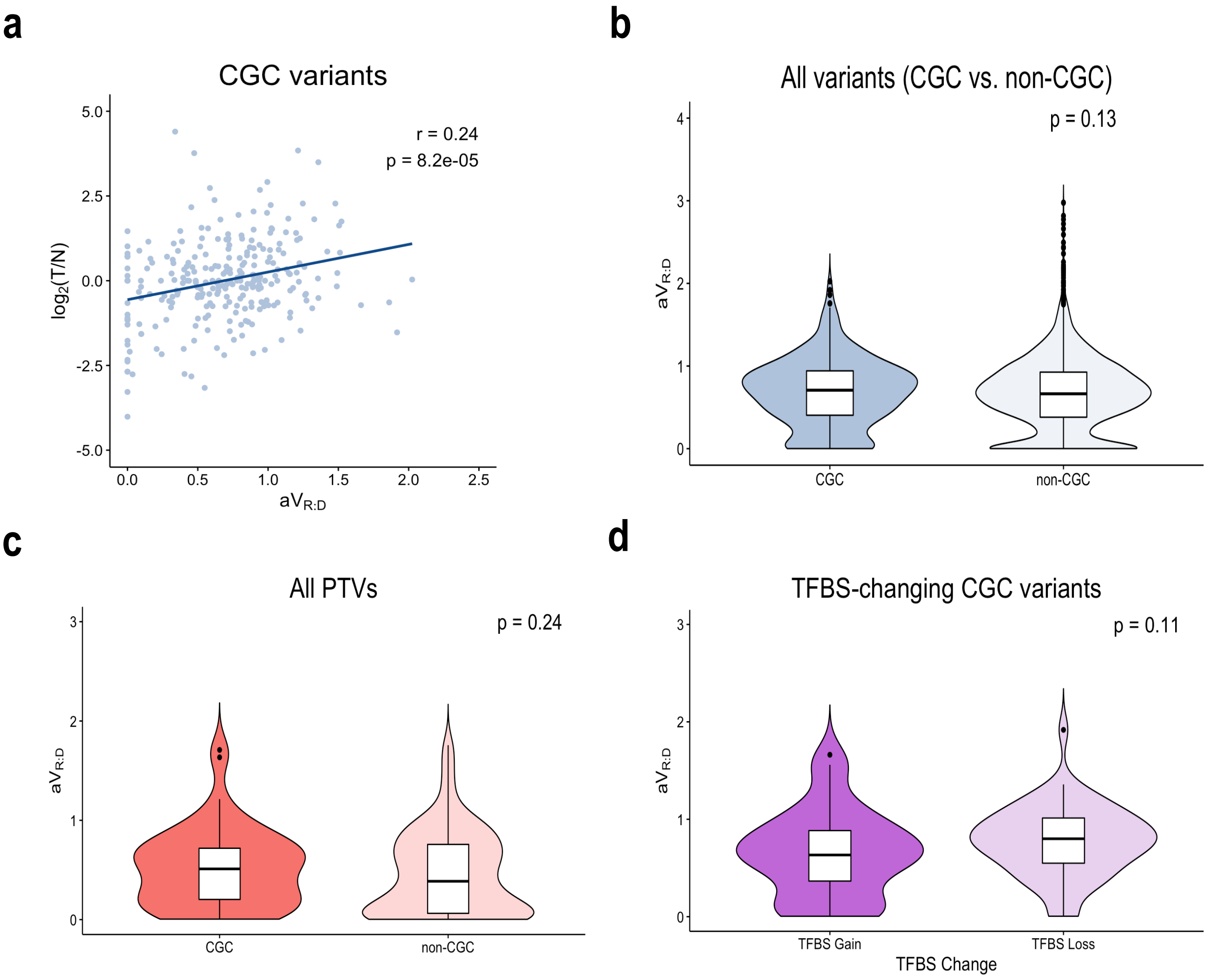

Supplementary Figure 5. Analyses of variant allele frequency adjusted through ABSOLUTE-assessed purity ($aV_{R:D}$). **a.** Correlation between variant allele fraction and gene expression change. **b**. Distribution of variant allele frequency of CGC- and non-CGC somatic variants . **c**. Distribution of variant allele frequency in PTVs in CGC and those in non-CGC. **d**. Distribution of variant allele frequency in somatic variants that generate a new TFBS and those that destroy an existing TFBS. The results are co-directional with the other purity adjusted estimations.

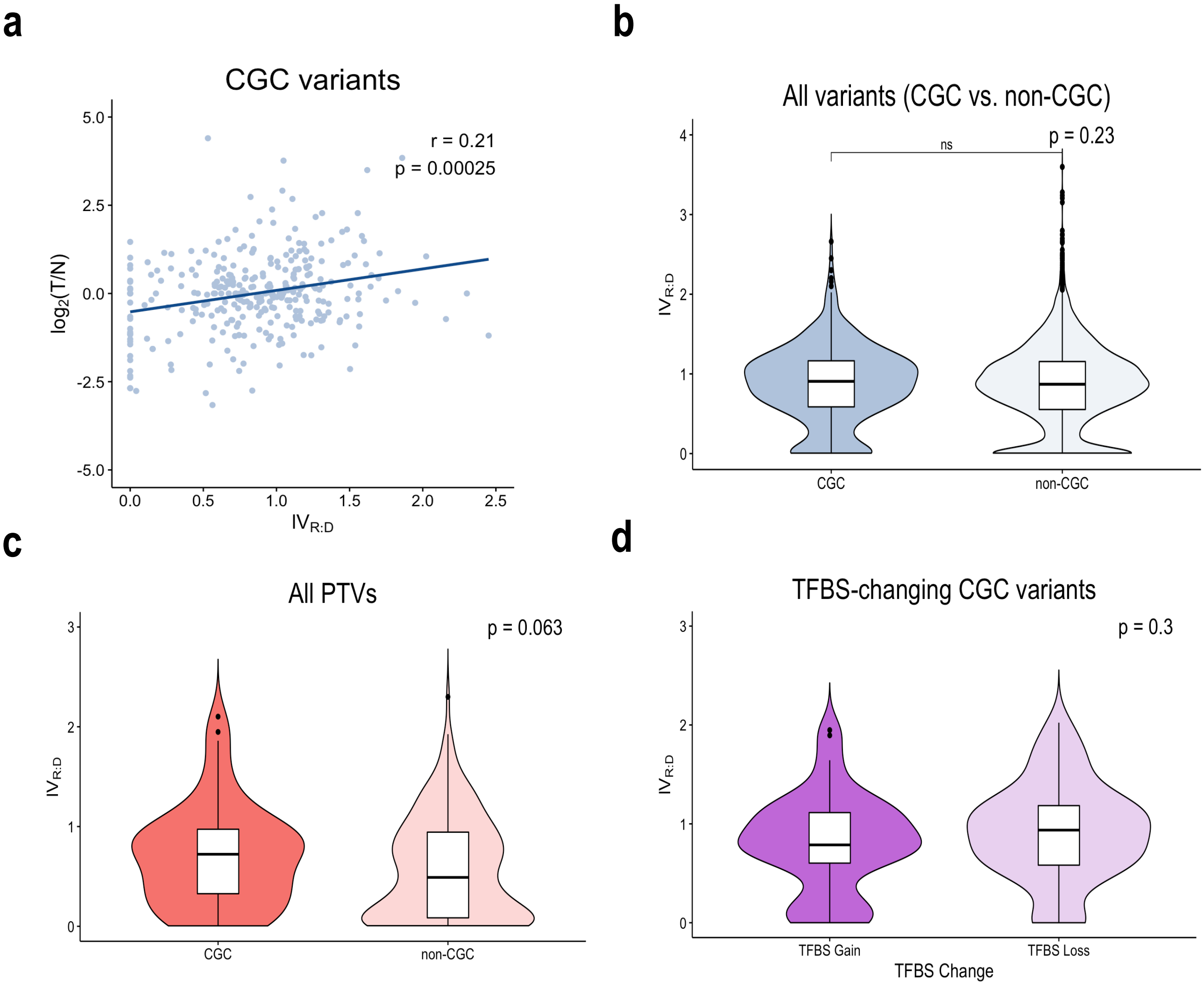

Supplementary Figure 6. Analyses of variant allele frequency adjusted through LUMP-assessed purity ($IV_{R:D}$). **a.** Correlation between variant allele fraction and gene expression change. **b**. Distribution of variant allele frequency of CGC- and non-CGC somatic variants . **c**. Distribution of variant allele frequency in PTVs in CGC and those in non-CGC. **d**. Distribution of variant allele frequency in somatic variants that generate a new TFBS and those that destroy an existing TFBS. The results are co-directional with the other purity adjusted estimations.

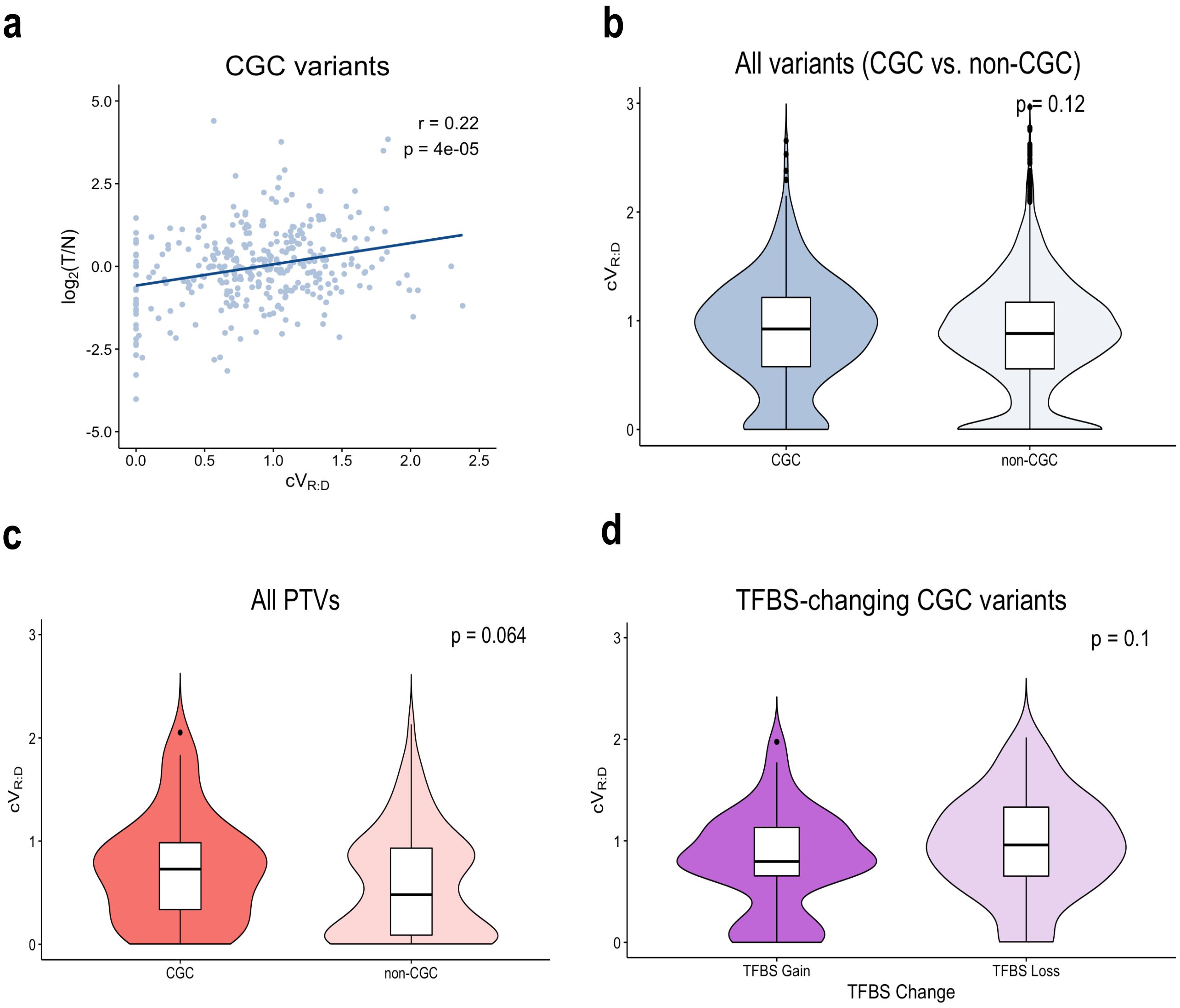

Supplementary Figure 7. Analyses of variant allele frequency (relative to DNA, $cV_{R:D}$) adjusted for purity using the Consensus Purity Estimation (CPE). **a.** Correlation between variant allele fraction and gene expression change. **b**. Distribution of variant allele frequency of CGC- and non-CGC somatic variants . **c**. Distribution of variant allele frequency in PTVs in CGC and those in non-CGC. **d**. Distribution of variant allele frequency in somatic variants that generate a new TFBS and those that destroy an existing TFBS. The results are co-directional with the other purity adjusted estimations.

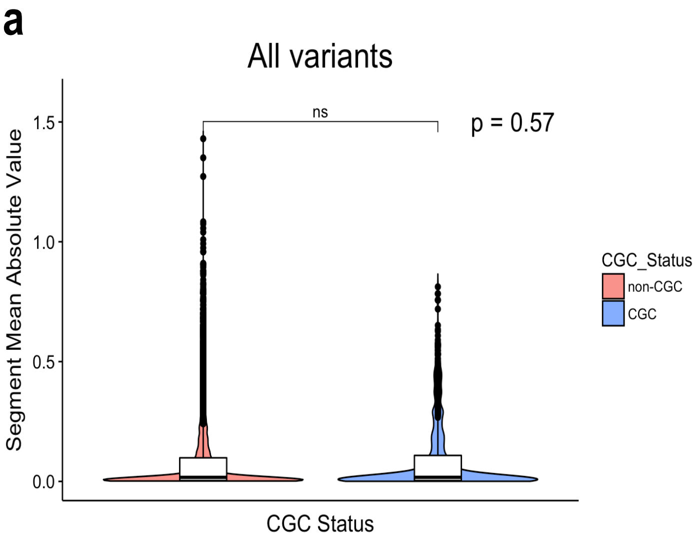

Supplementary Figure 8. Distribution of the absolute values of the segment mean (log2(copy-number/2)) assessments for CNAs in the loci of the SNVs analyzed in our study between CGC and non-CGC genes in the entire dataset (**a**), in the subsets of SOM-E (**b**) and SOM-L (**c**) somatic variants, and in the subsets of SOM-E PTVs (**d**) and SOM-L PTVs (**e**). None of these comparisons showed significantly different distribution of the segment mean absolute values between SNVs in CGC and non-CGC genes.

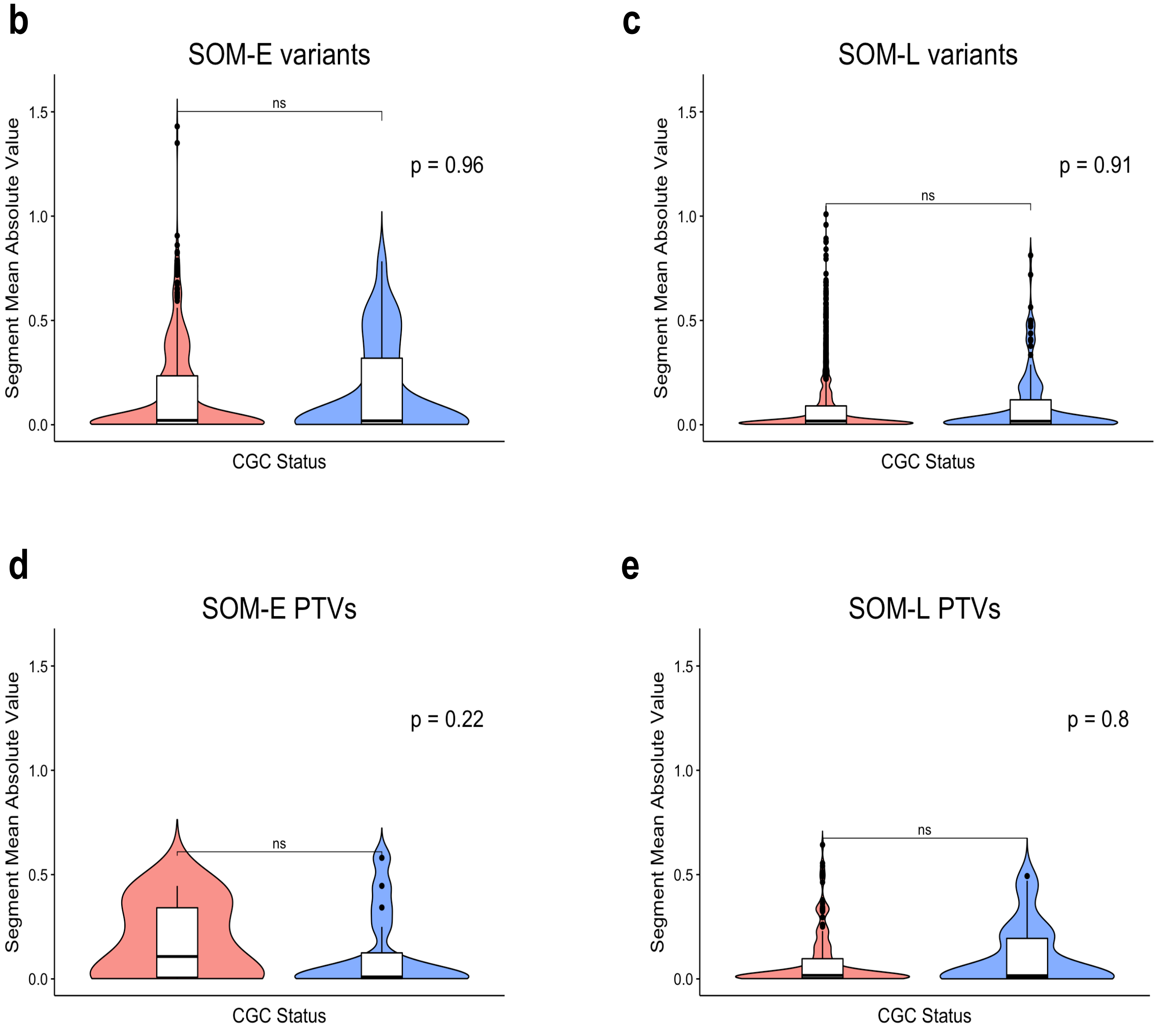

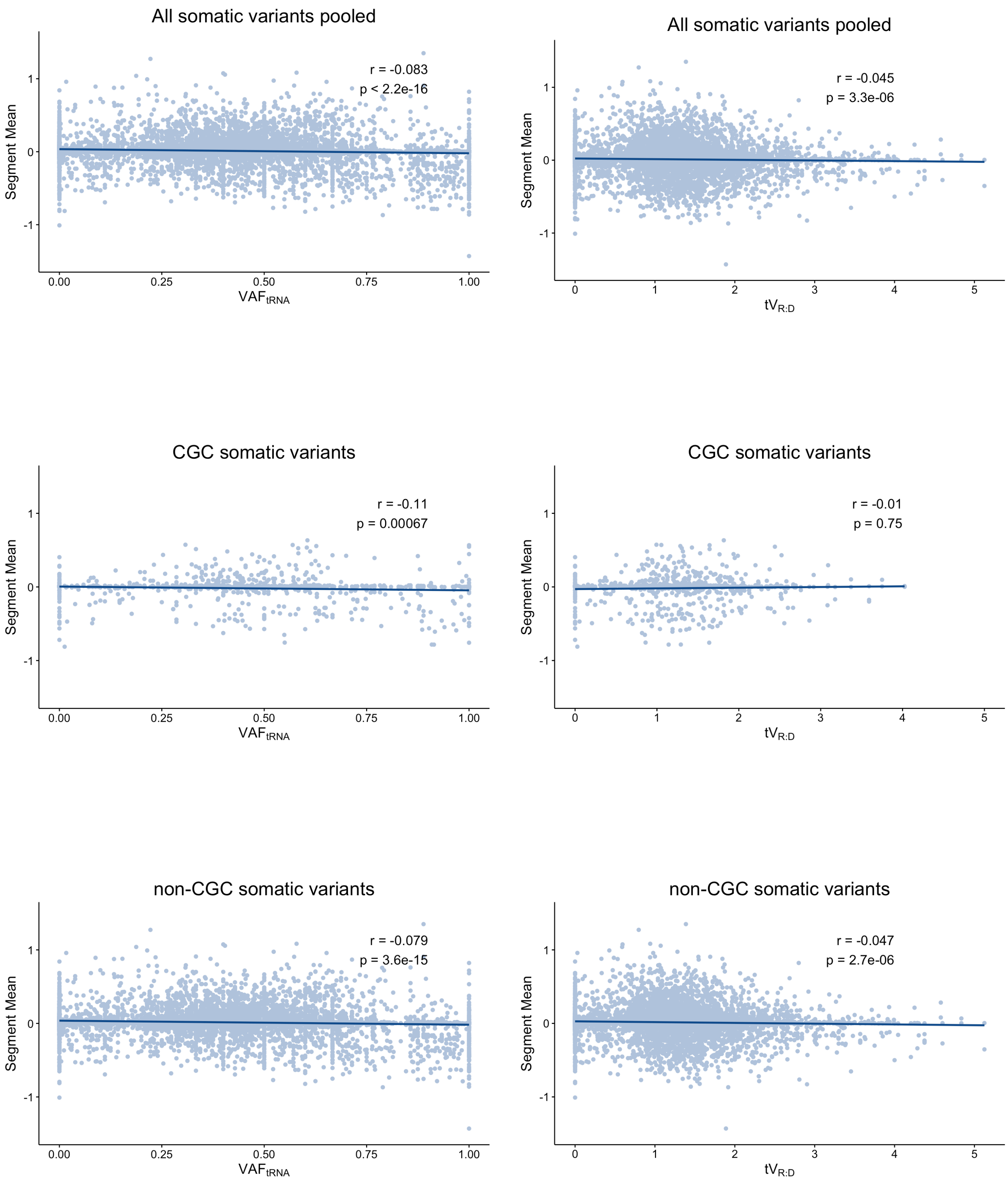

Supplementary Figure 9a. Correlation between variant allele frequency (VAF_{tRNA}, left, $tV_{R:D}$, right) in all somatic variants (top), CGC somatic variants (middle) and non-CGC somatic variants (bottom) with segment mean (log2(copy-number/2)) assessments for CNAs in the SNV-harboring loci. No positive or negative correlation was observed in any of the subsets.

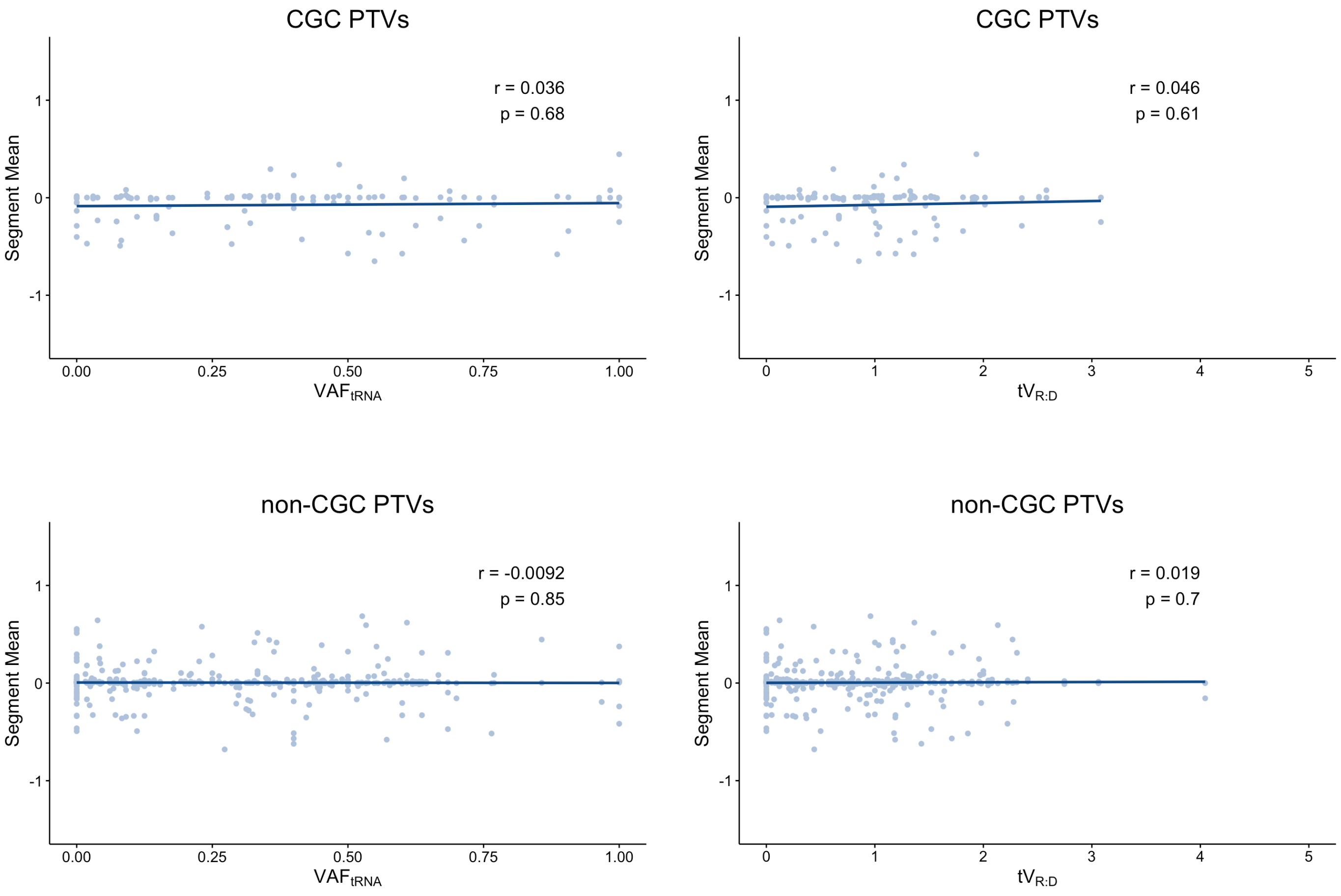

Supplementary Figure 9b. Correlation between variant allele frequency (VAF_{tRNA}, left, $tV_{R:D}$, right) in somatic PTVs in CGC genes (top) and non-CGC genes (bottom) with segment mean (log2(copy-number/2)) of the harboring loci. No positive or negative correlation was observed in any of the subsets.
